# Synthesis, and docking studies of novel heterocycles incorporating the indazolylthiazole moiety as antimicrobial and anticancer agents

**DOI:** 10.1038/s41598-022-07456-1

**Published:** 2022-03-02

**Authors:** Nadia T. A. Dawoud, Esmail M. El-Fakharany, Abdallah E. Abdallah, Hamada El-Gendi, Doaa R. Lotfy

**Affiliations:** 1 Chemistry Department, Faculty of Science, Girl’s, Al-Azhar University, Nasr City, Cairo Egypt; 2 Protein Research Department, Genetic Engineering and Biotechnology Research Institute GEBRI, City of Scientific Research and Technological Applications, New Borg El Arab, Alexandria 21934 Egypt; 3 Pharmaceutical Medicinal Chemistry & Drug Design Department, Faculty of Pharmacy (Boys), Al-Azhar University, Nasr City, Cairo 11884 Egypt; 4 Bioprocess Development Department, Genetic Engineering and Biotechnology Research Institute, City of Scientific Research and Technological Applications, New Borg El Arab, Alexandria 21934 Egypt

**Keywords:** Biochemistry, Chemical biology, Drug discovery, Chemistry

## Abstract

The current study was directed toward developing a new series of fused heterocycles incorporating indazolylthiazole moiety. The newly synthesized compounds were characterized through elemental analysis and spectral data (IR, 1H-NMR, 13C-NMR, and Mass Spectrometry). The cytotoxic effect of the newly synthesized compounds was evaluated against normal human cells (HFB-4) and cancer cell lines (HepG-2 and Caco-2). Among the synthesized compounds, derivatives **4**, and **6** revealed a significant selective antitumor activity, in a dose-dependent manner, against both HepG-2 and Caco-2 cell lines, with lower risk toward HFB-4 cells (normal cells). Derivative **8** revealed the maximum antitumor activity toward both tumor cell lines, with an SI value of about 26 and IC50 value of about 5.9 μg/mL. The effect of these derivatives (**8**, **4**, and **6**) upon the expression of 5 tumor regulating genes was studied through quantitative real-time PCR, where its interaction with these genes was simulated through the molecular docking study. Furthermore, the antimicrobial activity results revealed that compounds **2**, **7**, **8**, and **9** have a potential antimicrobial activity, with maximum broad-spectrum activity through compound **3** against the three tested pathogens: *Streptococcus mutans*, *Pseudomonas aeruginosa*, and *Candida albicans*. The newly prepared compounds also revealed anti-biofilm formation activity with maximum activity against *Streptococcus mutans*, *Pseudomonas aeruginosa*, and *Candida albicans*, respectively.

## Introduction

Cancer, one of the fundamental challenges for human health, represents the second causative agent of human mortality, following cardiovascular diseases^1^. Recent studies have shown that hepatocellular carcinoma and colorectal cancer are among the most common and fatal cancer types^2,3^. Hepatocellular carcinoma (HCC) is a worldwide health care issue that accounts for a high rate of mortality^2,4^. Many factors are associated with HCC development, including chronic infections with hepatitis viruses (hepatitis B and C), aflatoxin exposure, obesity, prolonged and/or heavy alcohol consumption^5,6^. Currently, the only FDA-approved medication for HCC treatment is Sorafenib (a multikinase inhibitor), which can only extend patient survival for a brief period^7^. Though the extrahepatic invasion of HCC outside liver cells is uncommon, growing evidence for the rare colon metastasis with HCC is treated only with surgical resection^8^. In the same context, colorectal cancer (CRC) characterized by a high mortality rate, is the second leading cause of death among cancer-affected patients^9^. The current procedures applied for cancer prevention and treatment including radiation, chemotherapy, and surgery are usually combined with immunosuppression for treated patients, which makes them susceptible to microbial infections^10^. In addition, none of the applied treatments satisfy the requirements for selectivity and high efficacy toward cancer cells and posttreatment side-effects are many and serious. As a result, ongoing research to develop highly selective new antitumor candidates with effective antimicrobial properties is a pressing demand toward an effective treatment for cancer patients^11^.

In the last few decades, anticancer drugs have been developed from many chemically synthesized compounds^11,12^. Heterocyclic derivatives, which contain at least two different elements, have received a great deal of attention in the development of pharmacologically active molecules and advanced organic materials^13,14^ and account for more than 75% of the materials currently used clinically^15^. Due to their broad biological and pharmacological applications, Sulfur, nitrogen, and/or oxygen-containing heterocyclic compounds such as thiophene and pyrazole are always of interest to medicinal chemists and researchers^16–18^. Thiophene has been dubbed the "wonder heterocycle" due to its diverse biological activities, which include anticancer^19–21^, antimicrobial^22–24^, antioxidant^25^, and anti-inflammatory^26^. The name “thiophene” was derived from the Greek words ‘theion’ for sulfur and ‘phaino’ for shining^27^. Tiquizium Bromides, Tipepidine, Tioconazole, and Citizolam among many marketed pharmaceutical drugs that contain thiophene nucleus^28–32^. Nevertheless, thiophene's low water solubility, combined with its hepatotoxicity, limited its wide widespread application and even forces the removal of many thiophene-containing medicines from the drug market^12^. On the other hand, Pyrazole is well known as a five-membered heterocyclic compound that has two neighboring nitrogen atoms, C_3_H_3_N_2_H. Pyrazole derivatives have been used for many years in agrochemicals as herbicides^33^ and in the pharmaceuticals field as antimicrobial^34^, anti-inflammatory^35^, anticonvulsant^36,37^, and anticancer^38^, with pyrazole's recent application as a selective analgesic and anti-inflammatory drug (COX-2 inhibitor) attracting more attention in medicinal chemistry^33^. With the recent advances in molecular hybridization strategies, new structures could be developed toward a novel antitumor drug with lower cytotoxicity. This strategy is based upon combining pharmacophoric moieties of various bioactive compounds to develop a new potent hybrid compound with higher activity compared to the parent’s structures^39^. According to the preceding information, the present study concerns the synthesis and characterization of novel furan, thiophene, pyrazole, pyran, and pyridine derivatives from indazolylthiazolidinone moieties. In addition, their biological potential, including anti-tumor and anti-microbial activities, was evaluated.

## Results and discussion

### Chemistry and characterization

The pressing demand for highly selective novel anticancer drugs forces continuous research to develop new compounds toward an effective treatment for cancer-affected patients^11^. The molecular hybridization strategy of combining the active moieties of different compounds represents a promising tool for developing new structures with higher biological activity than their starting precursors^39^. In order to continue our search for novel heterocyclic chemistry-based anticancer agents, we used indazolylthiazolidin-4-one (**1**), which was prepared using the reported procedure by reacting indazole-2-carbothioamide and chloroacetic acid in dimethylformamide^40^. Reactivity of the thiazolidine moiety was tested by the reaction of compound **1** with different reagents, as depicted in (see Electronic Supplementary Material Fig. 13). The solution of compound **1** in ethanol containing a catalytic amount of trimethylamine was treated with sulfanilamide^41^, and the mixture was heated at reflux temperature to give the corresponding amino benzenesulfonamide derivative **2**, which reacted with arylidenemalononitrile^42^ to give the pyridine derivative **3**. IR, mass spectrometry, and ^13^C-NMR spectra were consistent with the proposed structures. (See, Electronic Supplementary Material Fig. S1a, b, and Fig. S2a, b and c).

Treatment of compound **1** with malononitrile in refluxing ethanol containing a catalytic amount of piperidine and/or in dioxane containing Et_3_N under reflux temperature yielded the corresponding substituted furothiazole derivative **4**. The proposed structure of derivative **4** was confirmed by an IR spectrum, which revealed absorption bands at 3334–3215 and 2191 cm^−1^, demonstrating the presence of NH2 and CN functions, respectively, with the concurrent disappearance of the carbonyl band. Its ^13^C-NMR showed a signal at 115.53 ppm due to heteroaromatic (> CN) (see Electronic Supplementary Material Fig. S3a, b, and c). Compound **1** is reacted with malononitrile and sulphur element in an ethanolic solution containing a few drops of piperidine to produce thienothiazole derivative **5** with a yield of 69%. The ^13^C-NMR spectrum of derivative **5** demonstrated the presence of characteristic signals at 115.00, 161.55, and 171.60 ppm for (heteroaromatic > CN, C=N, and S–C–N) (see Electronic Supplementary Material Fig. S4a, b, and c).

When compound **1** was refluxed with cyanoacetic acid hydrazide and elemental sulfur in dimethylformamide containing a catalytic amount of piperidine for 12 h, thienothiazole derivative **6** obtained in yield 52%. The structures of the latter products were established based on the appearance of NH and NH2 absorption bands in the 3440, and 3324–3193 cm^−1^ regions and a nitrile function at 2190 cm^−1^ with the absence of the band corresponding to the ethenone carbonyl group (COCH2) in compound (**1**) in their IR spectra. The ^1^H-NMR spectra of **5**, and **6** in DMSO-d6 are characterized by singlet signal in the regions of 6.95 ppm and 7.80 ppm, which indicates the presence of thiophene moiety NH2 protons exchangeable with D_2_O. In addition to the characteristic singlet signal assigned to hydrazide NH proton at 9.60 ppm, and another singlet signal at 4.30 ppm for hydrazide NH2 protons exchangeable with D_2_O) in case of compound **6** (see Electronic Supplementary Material Fig. S5a and b).

The synthesis of compounds **5** and **6** takes place via the following mechanism:
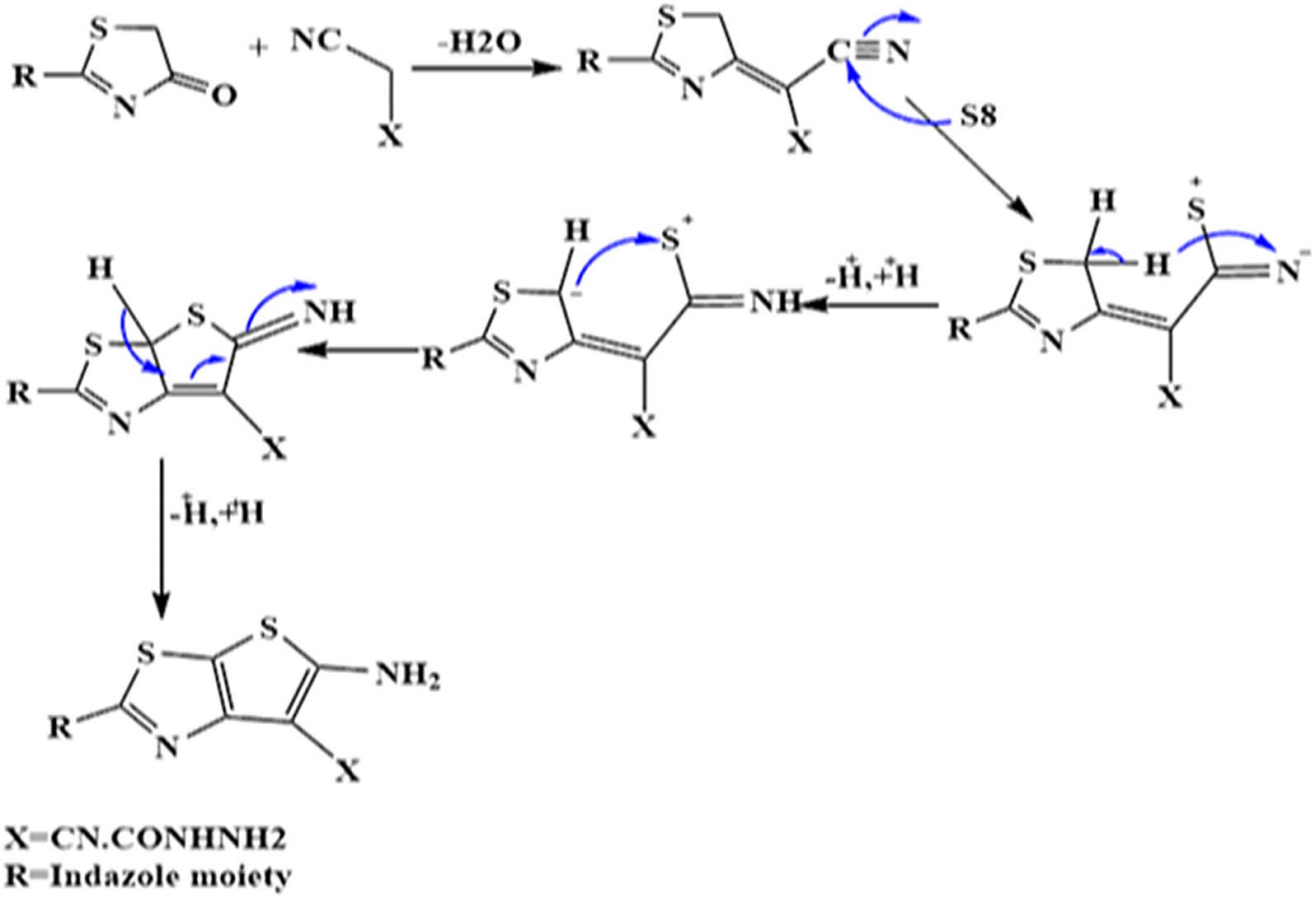


The proposed mechanism and pathway for conversion of indazolylthiazolidin-4 (compound **1**) into the corresponding derivatives **5**, and **6**.

Refluxing of compound **1** with ethyl cyanoacetate in dioxane containing a catalytic amount of piperidine^43^ yields the thiazolopyran derivative **7**. The structure of **7** was substantiated by the IR spectrum, which displayed a characteristic band at 1721 cm^−1^ assignable to the CO group of –lactone. Its ^1^H-NMR spectrum displayed singlets at 3.88 ppm attributable to the CH2 pyran protons and 8.40 ppm for C=NH (D_2_O exchangeable) (see Electronic Supplementary Material Fig. S6a, b, and c). The pyrazolo derivative **8** is obtained by the reaction of compound **1** with cyanoacetic hydrazide in boiling ethanol containing drops of Et_3_N^44^. The IR spectrum is assigned to the NH and CN groups, as well as the absence of the CO group. The ^1^H-NMR spectrum displayed a broad singlet at 3.39 ppm for CH2CN group protons with the presence of the characteristic absorption band at 2213 cm^−1^ due to the CN group in its IR spectrum (see Electronic Supplementary Material Fig. S7a and b).

The solution of compound **1** in dioxane in the presence of anhydrous K_2_CO_3_^45^ treated with ethyl chloroacetate and carbon disulfide in a 1:1:1 molar ratio was then heated at temperature reflux to yield the thienothiazole derivative **9**. The structure of product **9** was in agreement with its spectral and analytical data. For example, the IR spectrum revealed characteristic bands at 1731–1201 and 1028 cm^−1^ corresponding to CO and C=S groups. Its ^1^H-NMR spectrum showed one triplet at 1.18 ppm, corresponding to CH3 protons, and one quartet for CH2 protons at 4.10 ppm (see Electronic Supplementary Material Fig. S8a and b).

### Bioactivity of the synthesized indazolylthiazole-based heterocyclic compounds

#### Antitumor activity

**Figure 1:**
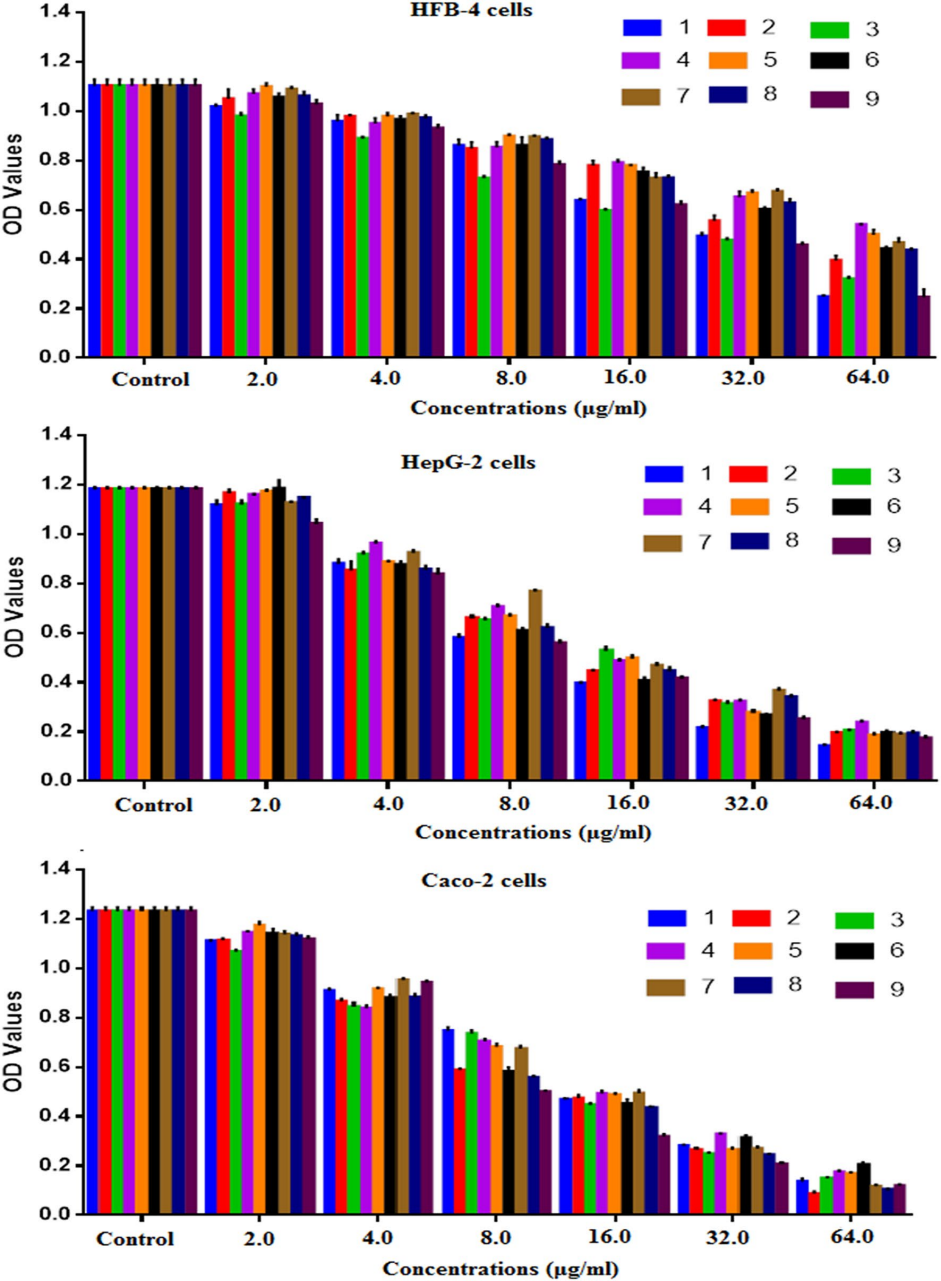
Effect of the prepared derivatives on the cell viability of the human normal (HFB-4) cells and human cancer (HepG-2 and Caco-2) cell lines after treatment for 48 h compared to untreated cells (expressed as triplicate values mean ± SEM).

The antitumor activity of the prepared derivatives was evaluated in vitro against HepG-2 (hepatoma) and Caco-2 (colon cancer) cells in comparison with normal human HBF-4 cells. Herein, the IC_50_ values of the synthesized derivatives against HFB-4 cells were calculated to range from 55.6 to 153.7 μg/mL (Table 1), indicating the significant safety of the prepared compounds toward normal human cells. On the other hand, the synthesized derivatives showed significant antitumor activity against both HepG-2 and Caco-2 cell lines with high SI values and low IC_50_ values. The results showed that the two tumor cell lines, HepG-2 and Caco-2, were nearly equally sensitive to the tested compounds. Moreover, the antitumor activity of the synthesized derivatives occurred in a dose-dependent way toward the tested cancer cells (Fig. 1). Our findings indicated that the synthesized derivative **8** showed superior antitumor activity toward both tested tumor cells indicated with an SI value of approximately 26, with very high selectivity toward tumor cells, as the IC_50_ values were nearly 5.9 μg/mL toward both HepG-2 and Caco-2 cell lines. Compound **8** followed by derivatives **6** (21.8 for HepG-2 and 25.6 for Caco-2) and **4** (18.1 for HepG-2 and 23 for Caco-2), as indicated in Table 1. The superior antitumor activity of compound **8** could be attributed to a thiazolyl–pyrazole moiety, which per the literature exhibits strong anticancer activity^43,46,47^. In addition, the presence of acetonitrile, incorporated into the pyrazole ring (at position 3), may reveal anticancer activity, as reported through other authors^44,45^.

**Table 1:** The antitumor activity of the prepared derivatives against HepG-2 and Caco-2 cells compared with normal human HBF-4 cells expressed in IC_50_ (μg/mL) and SI values.

Comp. No.	Cells				
	HFB-4	HepG-2		Caco-2	
	IC_50_	IC_50_	SI	IC_50_	SI
**2**	110.8 ± 1.54	6.37 ± 0.36	17.39 ± 0.24	6.06 ± 1.26	18.28 ± 0.25
**3**	55.59 ± 1.58	7.72 ± 0.38	7.20 ± 0.20	7.74 ± 0.81	7.18 ± 0.20
**4**	142.3 ± 1.48	7.88 ± 0.10	18.06 ± 0.19	6.18 ± 1.14	23.03 ± 0.24
**5**	97.66 ± 2.41	7.57 ± 0.36	12.90 ± 0.32	7.59 ± 0.24	12.87 ± 0.32
**6**	133.2 ± 4.18	6.12 ± 0.19	21.75 ± 0.03	5.21 ± 0.22	25.59 ± 0.03
**7**	104.8 ± 6.20	9.53 ± 0.32	10.99 ± 0.65	9.20 ± 0.27	11.39 ± 0.67
**8**	153.7 ± 1.76	5.86 ± 0.34	26.23 ± 0.30	5.89 ± 0.38	26.08 ± 0.30
**9**	65.65 ± 0.29	5.23 ± 0.25	12.55 ± 0.06	5.95 ± 0.10	11.04 ± 0.05

**Figure 2:**
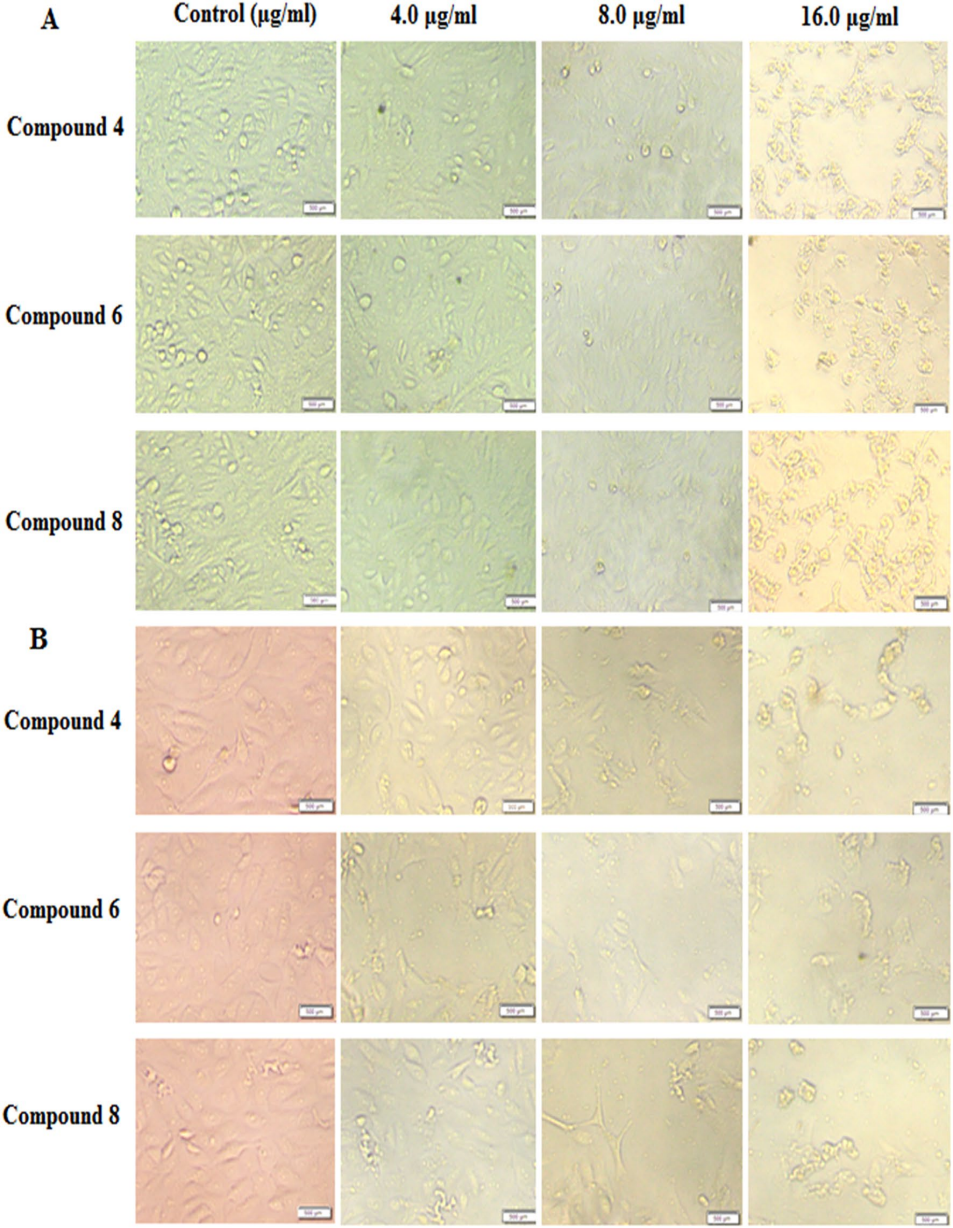
In vitro effect of the prepared derivatives on the morphological alterations of HepG-2 cells (**A**) and Caco-2 cells (**B**) as observed under a phase-contrast microscope. Both HepG-2 and Caco-2 cells were treated with synthetic compounds (**4**, **6**, and **8**) at various concentrations for 48 h and compared to untreated cells (control).

The proportional morphological changes of HepG-2 and Caco-2 cells upon treatment with the three potent antitumor derivatives (**4**, **6**, and **8**) at different concentrations (4–16 μg/mL) were studied in a live-cell mode by inverted microscopy, and the effect of these derivatives on the tested tumor cells was elucidated. The obtained images show that compounds **4**, **6**, and **8** enhance obvious cell damage and stimulate a clear alteration of the cell morphology in a dose-dependent way (Fig. 2). These cytotoxic modifications involved nuclear condensation and cell shrinkage, with some blabbing effects. Based on these observed results, the antitumor activity of the tested compounds seems to be enhanced by apoptotic molecular mechanisms.

#### Evaluation of the effect of the newly synthesized derivatives on some tumor gene expression

**Figure 3:**
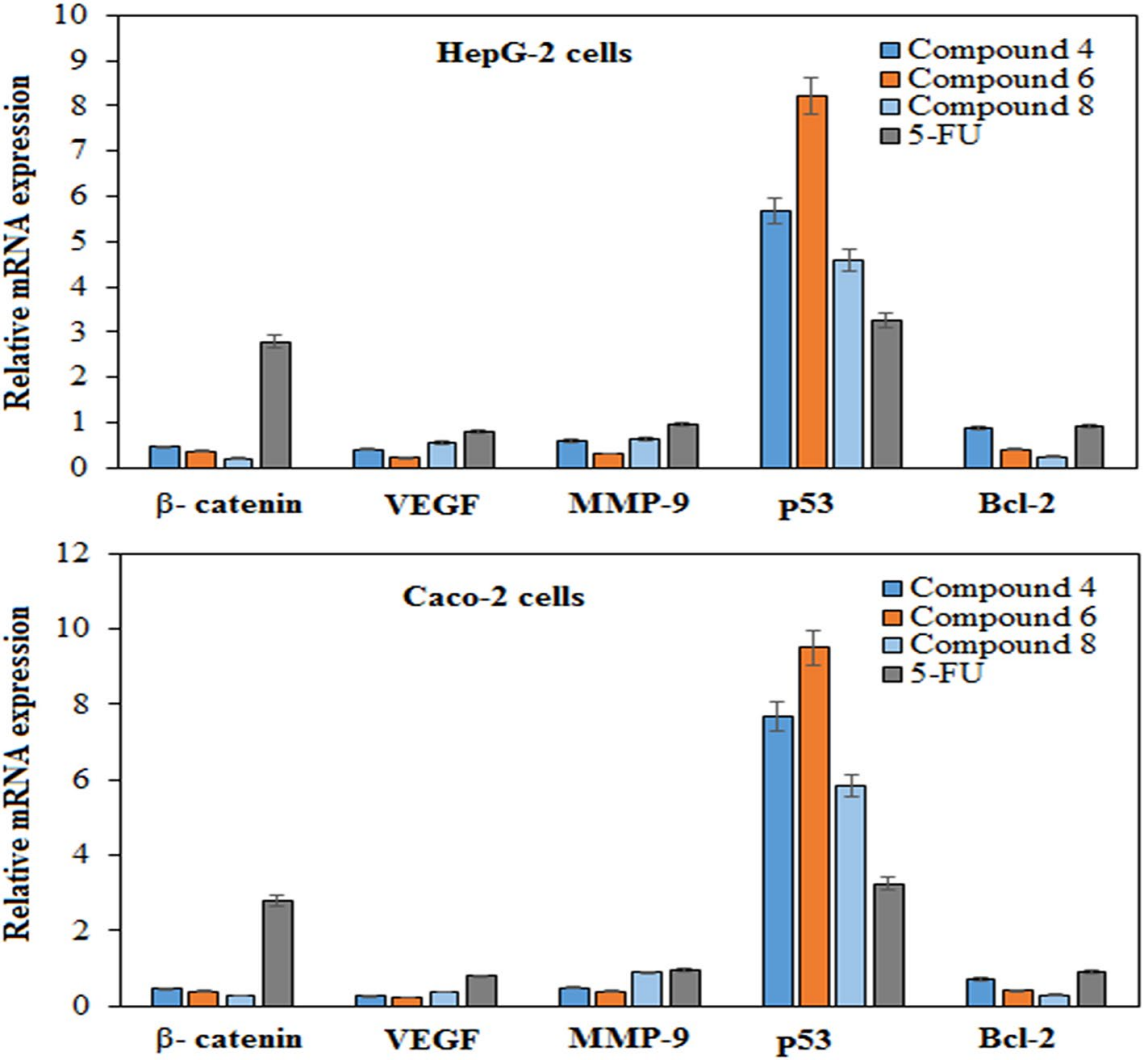
Evaluating the relative changes in mRNA expression levels of five key genes (β-catenin, VEGF, MMP-9, p53, and Bcl-2) in HepG-2 and Caco-2 cells treated with potent antitumor synthesized compounds compared to 5-FU standard anticancer drug.

The effect of the potent synthesized derivatives (**4**, **6**, and **8**) on five tumor regulating genes (β-catenin, VEGF, MMP-9, p53, and Bcl-2) was evaluated in both Caco-2 and HepG-2 cells using qRT^_^PCR and compared to 5-FU as a standard antitumor drug. Compounds **4**, **6**, and **8** significantly downregulated the gene expression of VEGF, MMP-9, and Bcl-2 compared to 5-FU and untreated cells (Fig. 3). These data reveal that downregulation of Bcl-2 expression was certainly stimulated by more than threefold compared to control cells. Additionally, they significantly suppressed the expression of the β-catenin gene in both HepG-2 and Caco-2 treated cells, in contrast to 5-FU, which stimulated its expression.

The β-catenin gene plays a major role in cell–cell adhesion in normal cells. Expression of this gene is under a strict regulation mechanism, where its overexpression is a main characteristic of cancer cells^48^. On the other hand, the expression level of p53 genes was dramatically upregulated by more than 2–8-fold in both treated HepG-2 and Caco-2 cells compared to control cells. p53 is an essential tumor-suppressor gene that triggers cell growth arrest and apoptosis, which are usually impaired in cancer cells^49^. Currently, p53 gene targeting is a promising tool for anticancer drugs^50^. The overexpression of p53 treated cells is in line with previous results (morphological changes study) showing that the applied derivatives induce apoptosis in cancer cells.

#### Cell cycle arrest analysis

**Figure 4:**
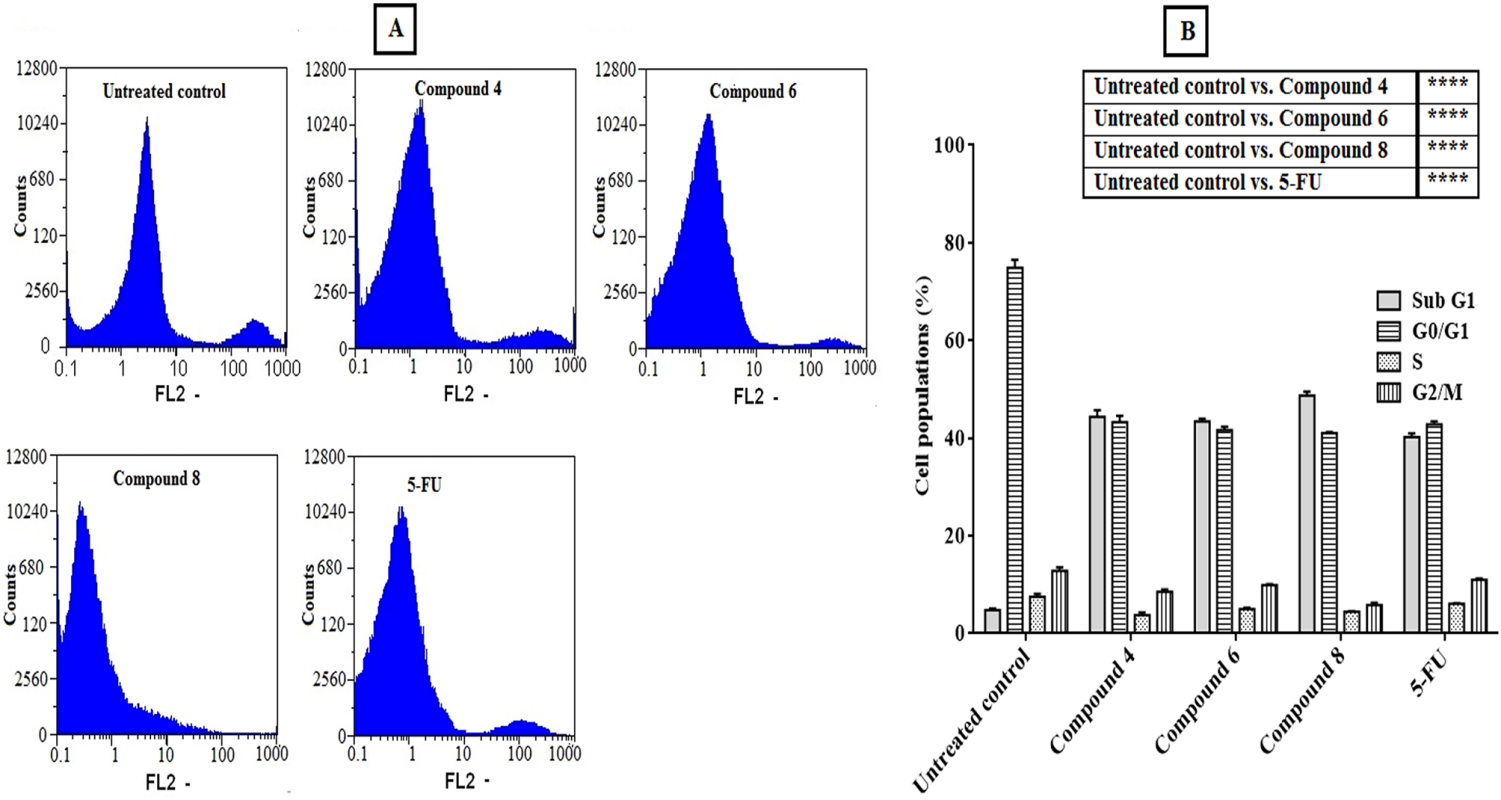
Cell cycle distribution analysis of Caco-2 cells treated with potent antitumor synthesized compounds compared to 5 FU standard anticancer drug for 48 h, (**A**) original flow charts of cell cycle diagrams, and (**B**) quantitative distribution of the treated cells in cell cycle phases compared with control (untreated) cells. Each bar represents the mean ± SEM (n = 3) and *p < 0.05, **p < 0.01, ***p < 0.001 and ****p < 0.0001 versus untreated control.

Arresting of cell cycle phases was studied for the treated Caco-2 cells with the most potent compounds to gain insight into their potential cellular mechanism to induce the anticancer effect. For this target, Caco-2 cells were treated with compounds **4**, **6**, and **8** for 48 h. Figure 4 shows the capability of these compounds to induce arresting of cell cycle distribution in both main checkpoints phases (G0/G1 and G2/M) of cell population growth. Our findings reveal that the apoptotic phase (sub-G1) population becomes detectable after treatment. In addition, the synthesis (S) phase was decreased after treatment with all compounds, especially in the case of treatment with compound **8**. Our significant results specify that the synthesized derivatives enhanced the cell cycle arrest of treated cells in both sub-G1 and S phases compared with untreated control.

#### Molecular docking

Molecular docking analysis has been one of the most basic and important strategies for drug design and discovery^51^. To this end, the interaction and affinity of the newly prepared potent anticancer compounds (**4**, **6**, and **8**) toward five proteins, MMP-9, p53, β-catenin, Bcl-2, and VEGF, were simulated through molecular docking. The proteins were chosen based on the effect of the new derivatives on the expression levels of several tumor-regulating genes, including MMP-9, p53, β-catenin, Bcl-2, and VEGF, as demonstrated in the biological discussion. The PDB IDs of these proteins used in the docking study are 4XCT, 3ZME, 1JDH, 2W3LL, and 2XAC. The crystal structures were downloaded and prepared for docking of our compounds (Tables 2, 3).

**Table 2:** Binding energies of the potent antitumor derivatives (**4**, **6**, and **8**) with the five examined proteins.

	4XCT	3ZME	1JDH	2W3L	2XAC
The ligand	− 6.53	− 6.82		− 5.23	
Compound **4**	− 5.74	− 4.45	− 5.92	− 4.95	− 4.34
Compound **6**	− 5.81	− 6.23	− 5.26	− 4.96	− 4.62
Compound **8**	− 5.88	− 5.15	− 5.79	− 4.91	− 4.08

**Table 3:** The residues involved in the interaction of the potent derivatives (**4**, **6**, and **8**) with the five selected proteins.

	MMP-9 (4XCT)	p53 (3ZME)	β-catenin (1JDH)	Bcl-2 (2W3L)	VEGF (2XAC)
The ligand	Ala189^a^	Asp228^a^		Tyr67^a^	
	His236^a^	Thr 230^b^			
	Leu188^b^	Cys229^b^			
Compound **4**	Ala189^a^	Cys220^a^	Asn430^a^	Glu95^a^	Gln27^a^
	Leu187^b^	Thr230^a^	Lys435^a^	Asp99^a^	
	His226^b^		Ser473^b^		
Compound **6**	Ala189^a^	Pro151^a^	Lys435^a^	Glu95^a^	Gln27^a^
	Leu187^b^	Cys220^a^	Ser473^a^	Asp99^a^	Gln55^a^
	His226^b^	Asp228^a^	Arg474^a^		Pro28^b^
			Arg469^b^		
Compound **8**	Tyr245^a^	Glu221^a^	Asn430^a^	Arg105^a^	Arg23^a&b^
	Arg249^a^		Lys435^a^		
	His236^b^		Arg474^a^		
			His470^b^		
			Arg515^b^		

^a^hydrogen bond and ^b^arene-H interaction.

**Figure 5:**
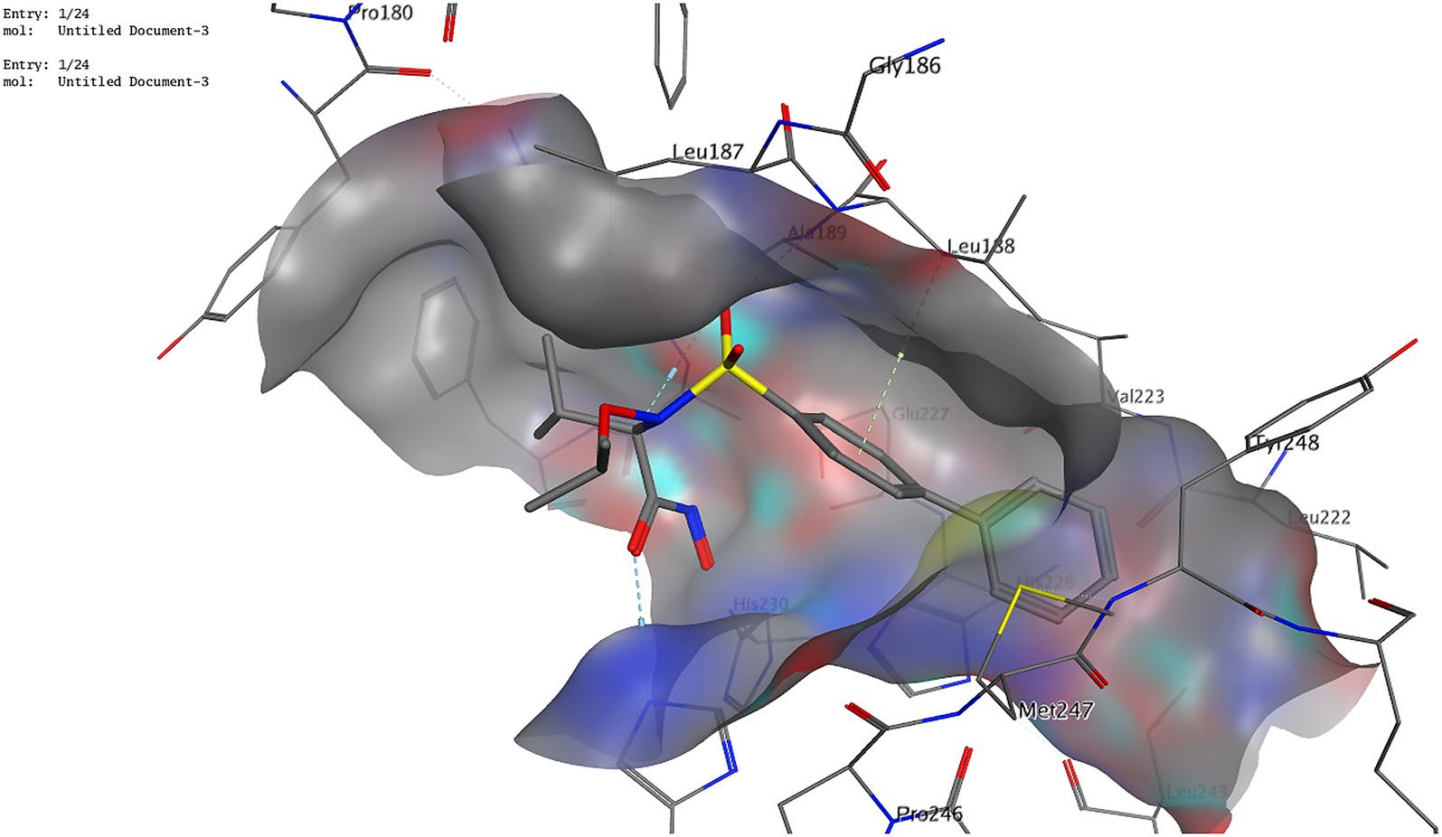
3D illustration of possible ligand interactions with the MM-9 protein (PDB ID 4XCT).

**Figure 6:**
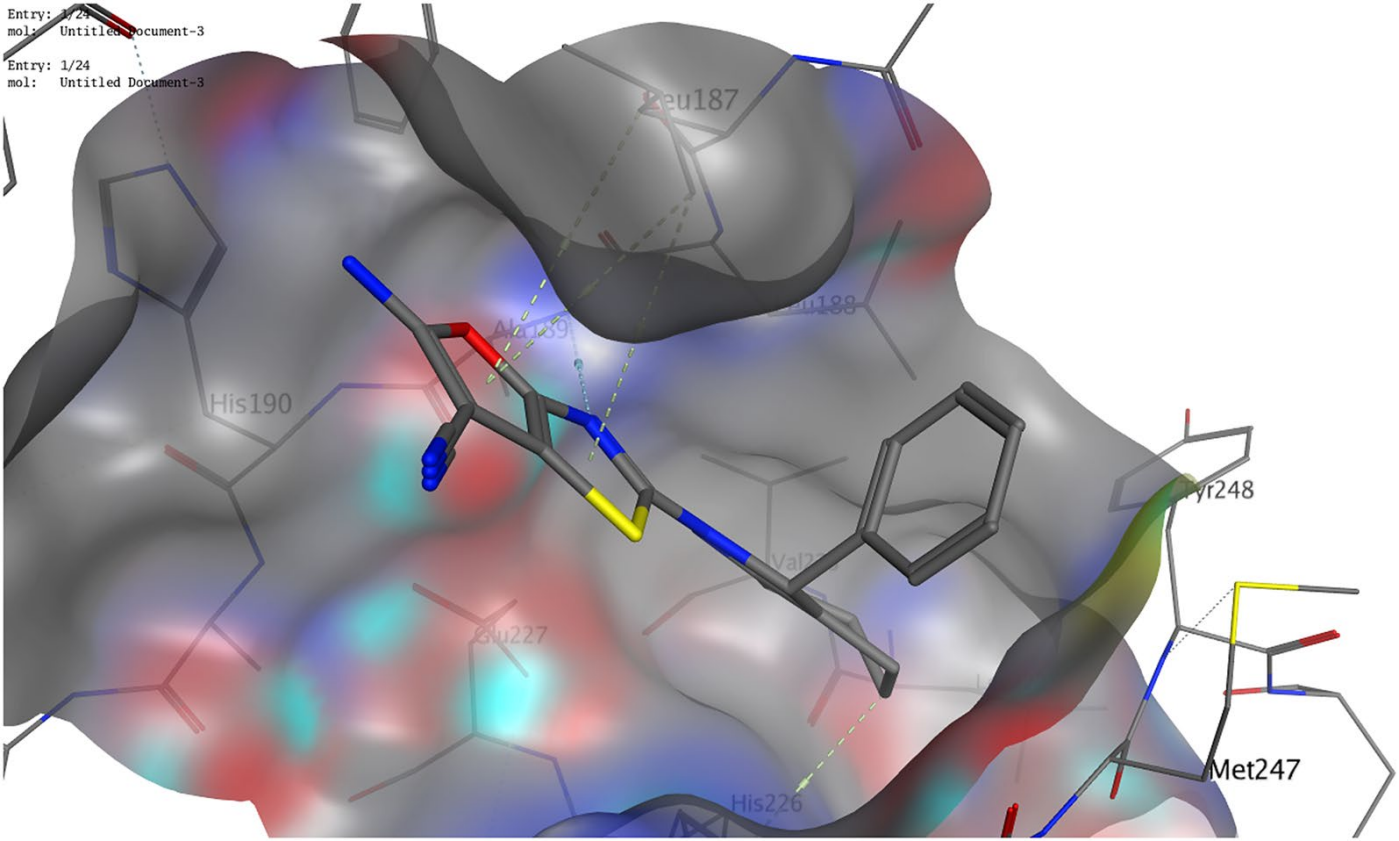
3D illustration of possible interactions of compound **4** with the MMP-9 protein (PDB ID 4XCT).

The obtained data showed that our compounds were able to bind effectively to MMP-9 (PDB ID: 4XCT). We can see three essential residues to which the ligand binds. Figure 5 reveals that the ligand formed two hydrogen bonds with Ala189 and His236 and one arene-H interaction with Leu188. Compound **4**, as well as compound **6**, showed a hydrogen bond with the essential amino acid Ala189. Moreover, two arene-H interactions with Leu187 and His226 were observed (Fig. 6), while compound **8** formed arene-H interactions with the essential residue His236. Furthermore, it exhibited two hydrogen bonds with Tyr245 and Arg249.

**Figure 7:**
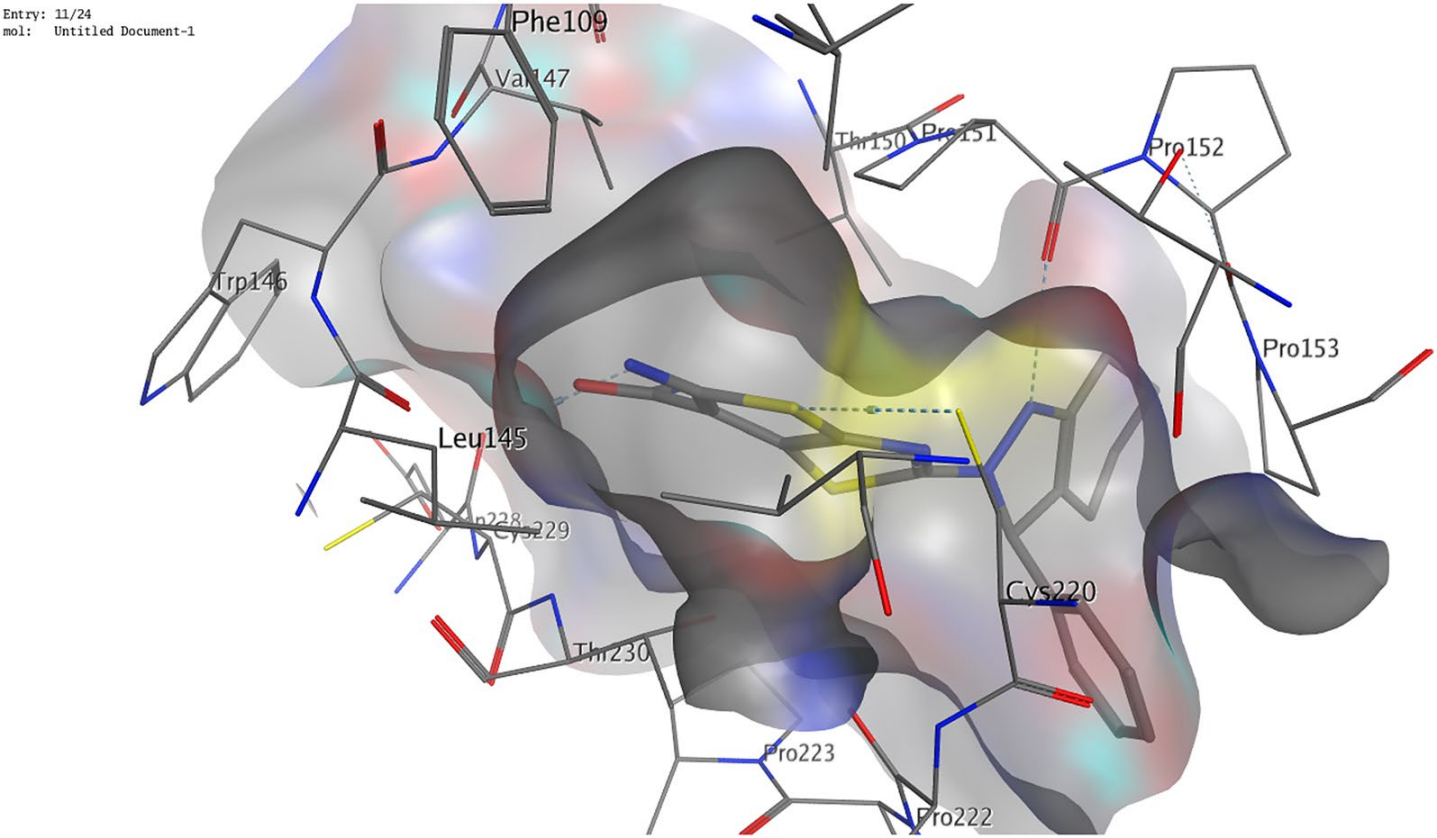
3D illustration of possible interactions of compound **6** with the p53 protein (PDB ID 3ZME).

**Figure 8:**
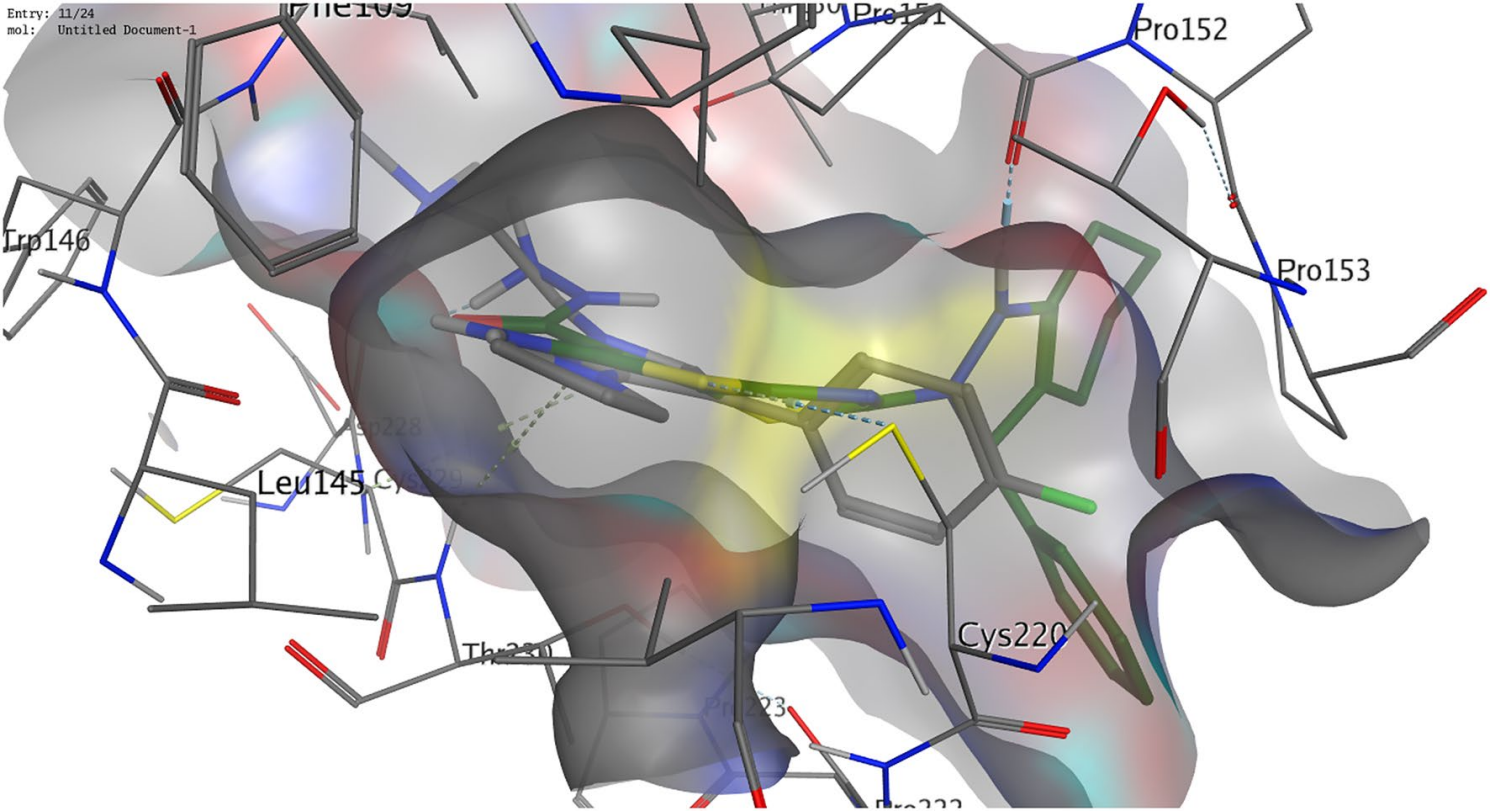
3D illustration of the overlay of compound **6** (green colored) and the ligand (brown colored) into p53 (PDB ID 3ZME).

Concerning p53 (PDB ID 3ZME), the ligand showed one hydrogen bond with Asp228 and two arene-H interactions with Thr230 and Cys229. Compound **6** was found to be the most promising candidate. It exhibited a correct binding mode. In addition to its ability to form a hydrogen bond with the essential amino acid residue Asp228, it showed two further hydrogen bonds with Pro151 and Cys220 (Figs. 7, 8). It also showed free binding energy (− 6.32 kcal/mol) nearly equal to that of the ligand (− 6.82 kcal/mol). Compound **4** exhibited the correct binding mode by forming two hydrogen bonds; one with the essential amino acid Thr230 and another with Cys220. Although compound **8** failed to form a hydrogen bond with an essential amino acid, it formed a hydrogen bond with Glu221.

**Figure 9:**
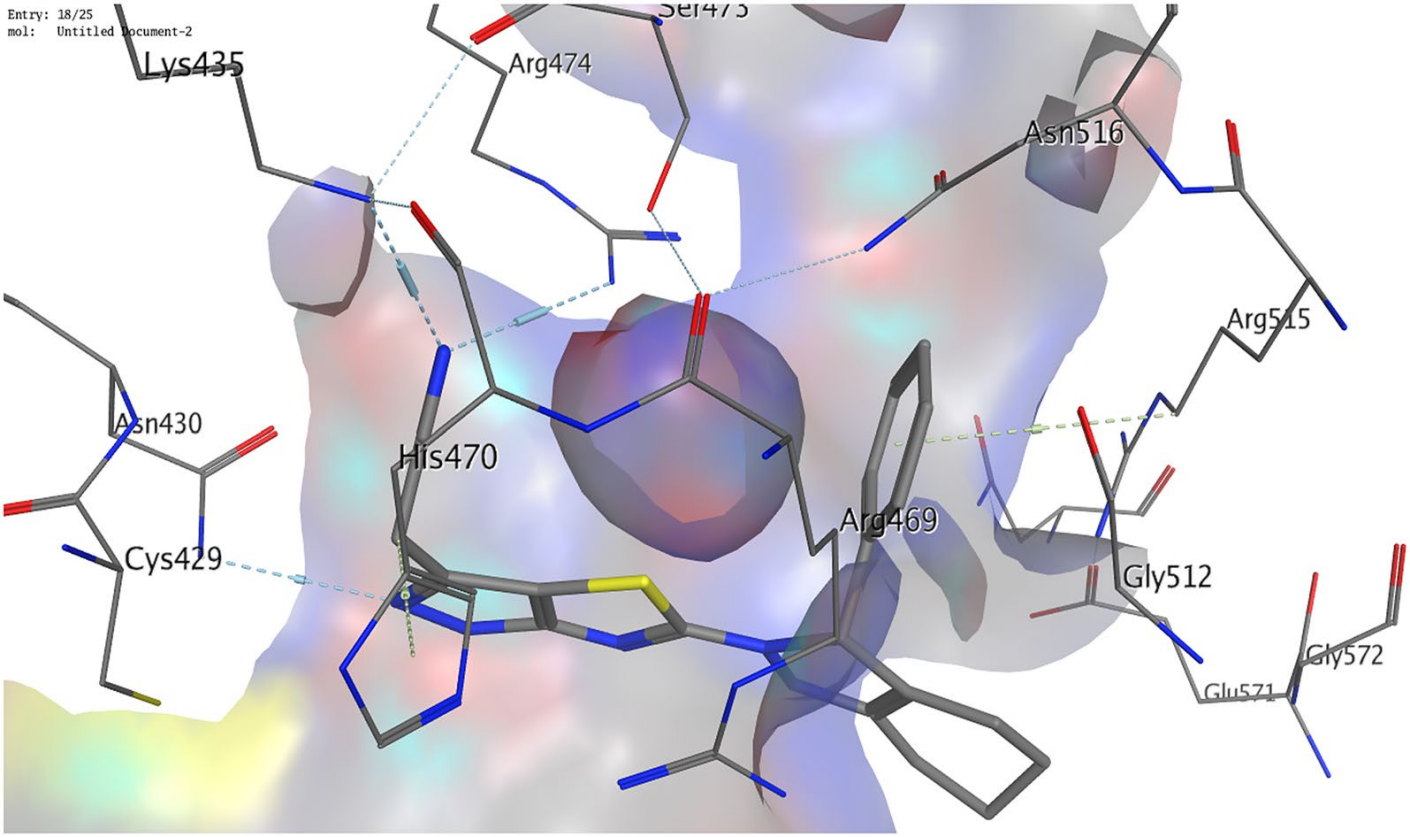
3D illustration of possible interactions of compound **8** with β-catenin protein (PDB ID 1JDH).

**Figure 10:**
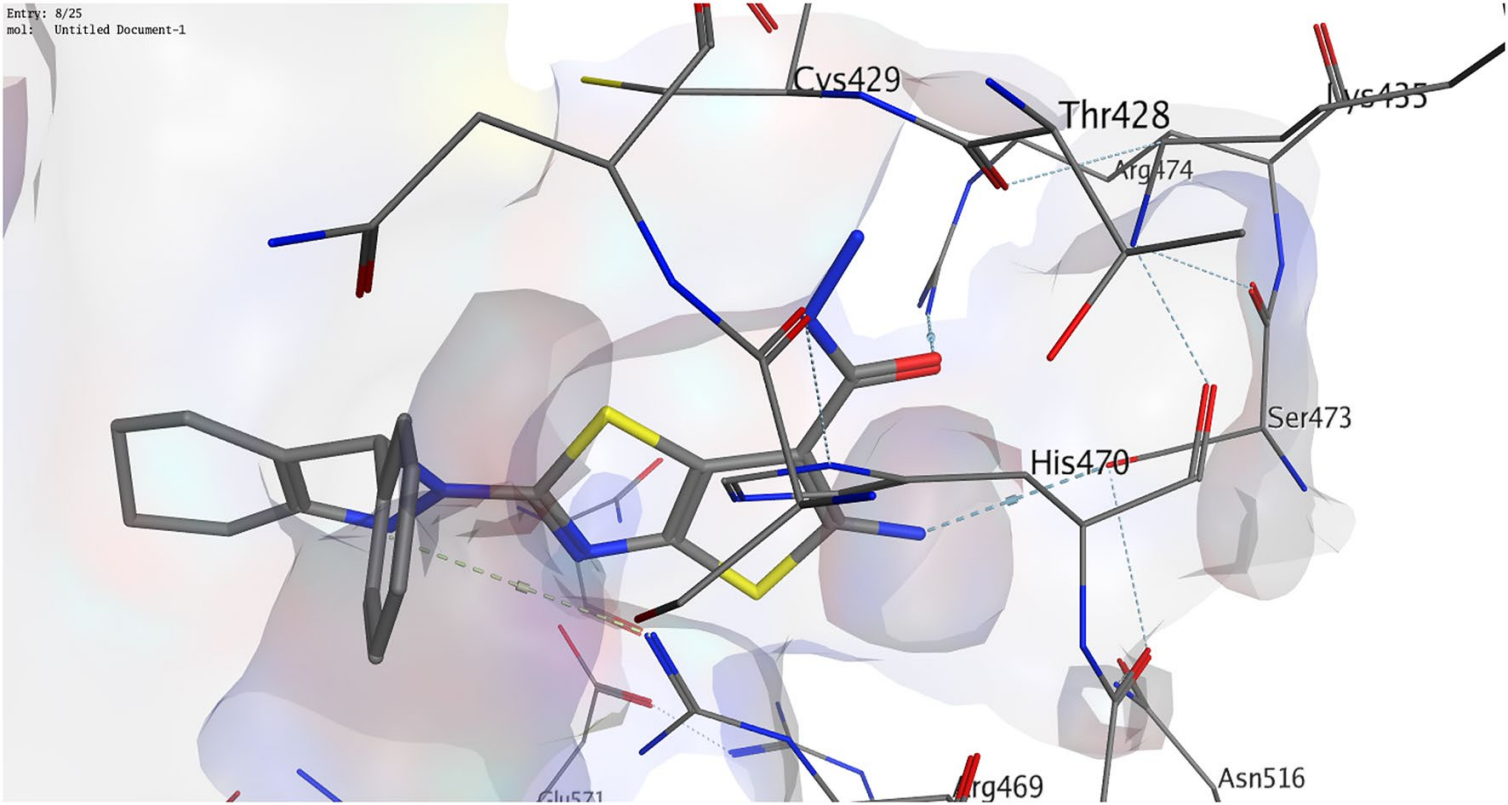
3D illustration of compound **6**'s possible interactions with β-catenin (PDB ID 1JDH).

Regarding β-catenin (PDB ID 1JDH), it was reported that the β-catenin residues His260, Asn261, Lys292, Ile296, Asp299, Tyr306, Gly307, Lys312, Lys335, Lys345, Arg376, Arg386, Asn387, Asn426, Cys429, Lys435, Cys466, His470, Arg474, and Lys508 are the residues that interact with TCF4 to form a complex^52,53^. Hence these residues are effective residues to which the inhibitor should bind. Our compounds showed interactions with more than one essential residue with binding energies ranging from − 5.2 to − 5.9 kcal/mol. Hence they are expected to be effective inhibitors of β-catenin. Compound **8** was the best as it showed interactions with three effective residues; Lys435, Arg474, and His470. It formed hydrogen bonds with Lys435 and Arg474 and an arene-H interaction with His470. Moreover, it further exhibited a hydrogen bond with Asn430 and an arene-H interaction with Arg515 (Fig. 9). Compound **6** showed two essential hydrogen bonds with the effective residues Lys435 and Arg474. Furthermore, a hydrogen bond was formed with Ser473 and an arene-H interaction with Arg469 as illustrated in Fig. 10. Compound **4** showed a hydrogen bond with the essential amino acid Lys435 and two hydrogen bonds with Asn430 and Ser473.

**Figure 11:**
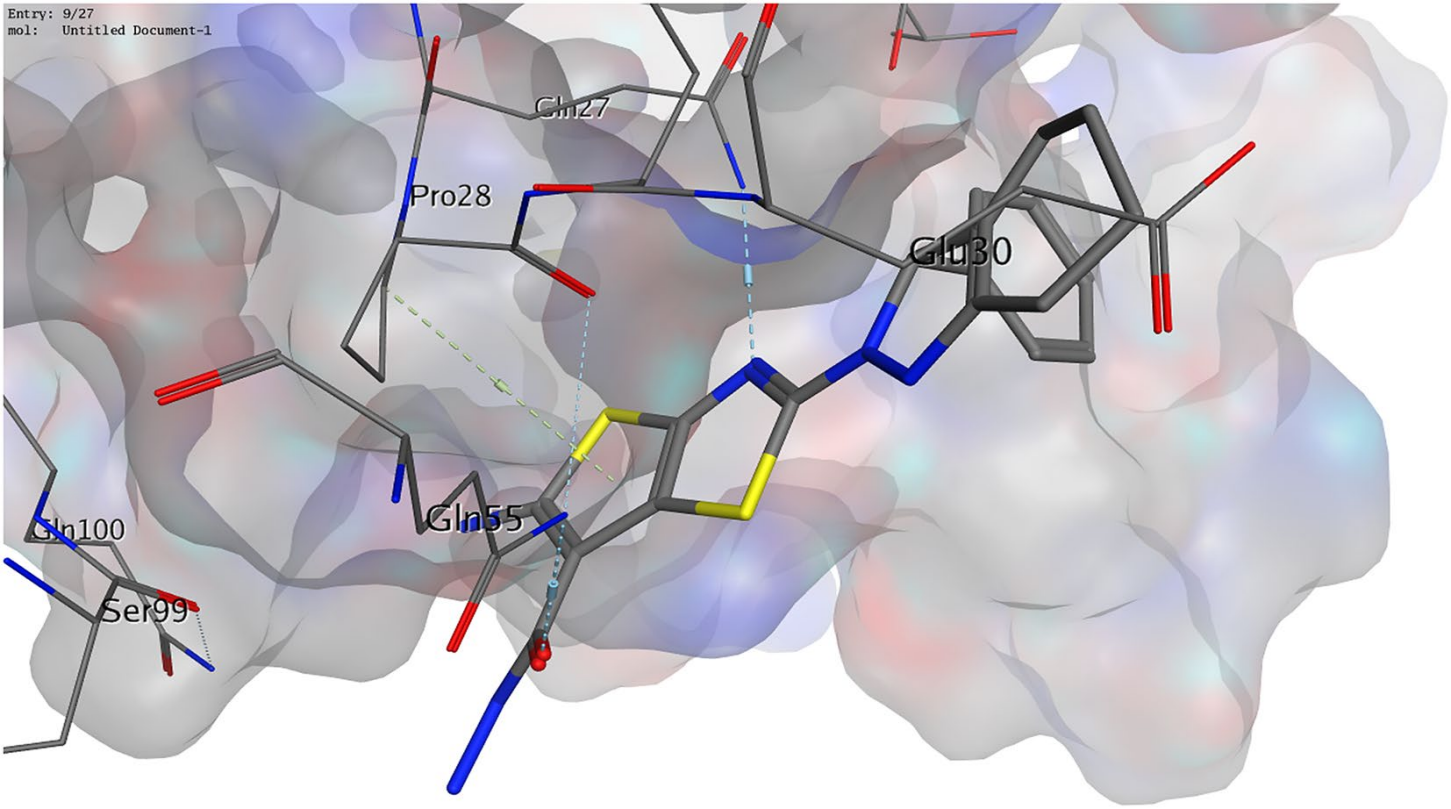
3D illustration of possible interactions of compound **6** with VEGF protein (PDB ID 2XAC).

Regarding Bcl-2 (PDB ID 2 W3LL), our compounds showed a binding manner that differed from that of the ligand. The ligand formed a hydrogen bond with Tyr67 to which none of our compounds bound, indicating that it is more likely that the studied compounds (**4**, **6**, and **8**) are not able to bind to this protein effectively. Finally, the crystal structure of VEGF (PDB ID 2XAC) suggests that Gln27 is one of the effective residues involved in the binding of VEGF to VEGFR^54^. Compound **4**, as well as compound **6**, showed a hydrogen bond to Gln27. Compound **6** also demonstrated a further hydrogen bond with Gln55 and arene-H interaction with Pro28 (Fig. 11), while compound **8** interacted with Arg23 only.

#### Antimicrobial efficacy of the prepared compounds

The prevalence of drug-resistant pathogens highlights the need for novel antimicrobials with lower resistance induction potential^55^. In this direction, the antimicrobial activity of a series of novel prepared compounds (**2–9**) was assessed against three pathogens, including *Streptococcus mutans, Pseudomonas aeruginosa,* and *Candida albicans* using a microliter-plate assay. The MIC results (Table 4) illustrated that derivatives **7** and **8** revealed reasonable antibacterial activity against *Streptococcus mutans,* with maximum inhibition activity by derivative **3** (MIC of 11.2 µg/mL), comparable to that of the ampicillin MIC (13.5 µg/mL). For Gram-negative bacteria (*Pseudomonas aeruginosa*), derivatives **2** and **7** revealed moderate activities with maximum inhibition through derivative **3** with a MIC of 18.29 µg/mL, which is nearly the same as the applied reference ciprofloxacin MIC of 18.7 µg/mL. The newly prepared compounds revealed low to medium antifungal activity against *Candida albicans* with a maximum antifungal activity through compound **3** (MIC of 40.74 µg/mL), representing 36% clotrimazole activity. In the scope of this study, among the newly peppered series, compound **3** revealed potent broad-spectrum antibacterial activity with moderate antifungal activity. The broad-spectrum antibacterial activity of compound **3** may be attributed to the incorporated thiazolopyridine moiety, which is accordant to^56^. Khidre and Radini, reported the broad-spectrum potency of the thiazolopyridine nucleus and its derivative against many human pathogens, hypothesized that, the possible mechanisms of the thiazolopyridine nucleus antimicrobial activity is through targeting and inactivating vital microbial enzymes such as DNA gyrase^57^.

**Table 4:** The antimicrobial activity of the newly synthesized compounds represented in MIC (µg/mL) toward *Streptococcus mutant, Pseudomonas aeruginosa,* and *Candida albicans.*

Compound	*Streptococcus mutant*	*Pseudomonas aeruginosa*	*Candida albicans*
Ampicillin	13.5 ± 0.234	–	–
Ciprofloxacin	–	18.7 ± 0.475	–
Clotrimazole	–	–	14.5 ± 1.25
**2**	49.71 ± 0.5849	40.13 ± 2.109	52.77 ± 0.7862
**3**	11.12 ± 0.4232	18.29 ± 2.114	40.74 ± 0.5511
**4**	34.41 ± 0.2374	60.76 ± 2.218	48.92 ± 1.054
**5**	43.52 ± 0.359	56.51 ± 2.191	59.02 ± 1.285
**6**	39.58 ± 0.3478	58.47 ± 2.267	57.3 ± 1.32
**7**	22.42 ± 0.3173	43.86 ± 2.042	58.18 ± 1.207
**8**	15.77 ± 0.2052	53.05 ± 2.183	56.17 ± 1.235
**9**	48.77 ± 0.2457	61.51 ± 1.832	46.61 ± 1.195

#### Microbial-biofilm inhibition activity

**Figure 12:**
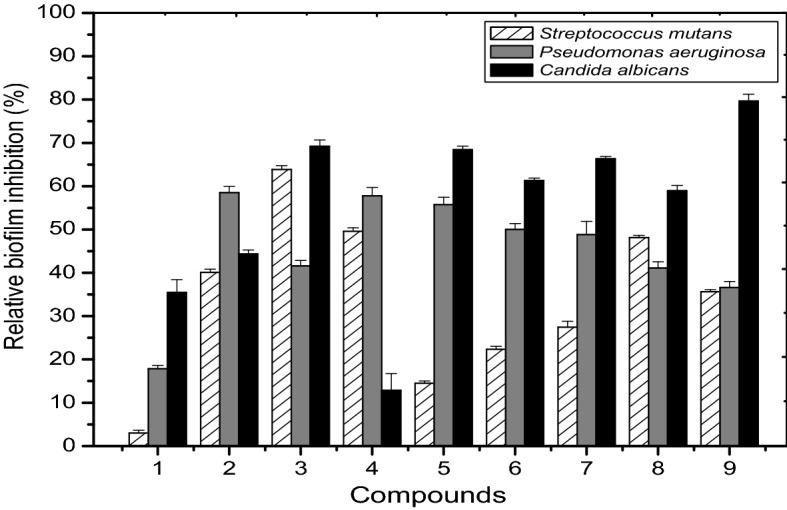
Efficacy of the precursor compound **1** and its newly prepared derivatives (compounds **2–9**) on microbial biofilm inhibition against three human pathogens: *Streptococcus mutant, Pseudomonas aeruginosa,* and *Candida albicans.* Compound **1** was included.

One of the key pathogenicity mechanisms for microbial pathogens is microbial-biofilm formation, which supports the persistence of infection^58^. As a result, compounds that interfere with biofilm formation improve microorganisms' susceptibility to therapeutic drugs. To this end, the efficacy of the newly prepared compounds (**2–9**) was evaluated for inhibition of microbial-biofilm formation using the TCP technique. The results (Fig. 12) revealed that compounds **3**, **4**, and **8** had antibiofilm formation activities against *Streptococcus mutans*, with maximum inhibition activity around 64% through compound **3** (Fig. 12), whereas compounds **2**, **3**, **4**, and **5** revealed high biofilm inhibition activity against *Pseudomonas aeruginosa* with a maximum biofilm inhibition through compound **2** of approximately 58.5%. In the case of *Candida albicans,* several preparations exerted antibiofilm activity, including compounds **3** (69%), **5** (68%), **6** (61%), and **7** (66%) with a maximum biofilm inhibition recorded through compound **9** of nearly 79%.

## Material and methods

### Chemistry and characterization

The melting point ranges were taken on a Gallenkamp electric melting point apparatus using the one-end open capillary method and were uncorrected. IR spectra were recorded (KBr discs) on a Shimadzu FT-IR 8201 PC spectrophotometer.^1^H-NMR and ^13^C-NMR spectra were recorded on a Bruker 300 spectrometer, using DMSO as the solvent and tetramethylsilane (TMS) as an internal reference. Mass spectroscopy was carried out using direct inlet unit (DI-50) of Shimadzu GC/MS-QP5050 A.

#### Synthesis of 4-(2-(3-phenyl-1,3,4,5,6,7-hexahydro-2H-indazol-2-yl)thiazol-4-yl) amino) benzenesulfonamide (2)

An ethanolic solution of compound **1** (10 mmol, 3 gm) containing a catalytic amount of triethylamine, and sulphanilamide (10 mmol, 1.8 gm) was heated under reflux for 12 h, cooled to room temperature (RT), and acidified by HCl. The solid formed was filtered off, washed with water, and purified by recrystallization from petroleum ether to give the required product **2** as a yellow powder, in a 53% yield; m. p: 120–123 °C; IR (KBr): ν (cm^−1^) 1154 SO2, 1598C=C, 1627C=N, 2933, 2857CH-Al, 3025CH-Ar, 3377, 3200 cm^−1^ of NH2; HRMS (m/z%): [M]^+^ calcd. for C_22_H_23_N_5_O_2_S_2_ 453(100); found 453(14.16); analysis (calc., found for C_22_H_23_N_5_O_2_S_2_): C(58.26, 58.07), H(5.11, 4.99), N(15.44, 15.29), S(14.14, 14.02).

#### Synthesis of 4-(5-amino-6-cyano-7-phenyl-2-(3-phenyl-1,3,4,5,6,7-hexahydro-2H-indazol-2-yl) thiazolo[4,5-b]pyridin-4(7H)-yl) benzenesulfonamide (3)

A mixture of 4-(2-(3-phenyl-1,3,4,5,6,7-hexahydro-2H-indazol-2-yl)thiazol-4-yl)amino) benzenesulfonamide **(2**) (10 mmol, 4.5 gm) and arylidenemalononitrile (10 mmol, 1.6 gm) in ethanol (20 mL) containing a catalytic amount of triethylamine (0.5 mL) was heated under reflux for 12 h, cooled to RT, and acidified by HCl. The solid formed was filtered off, washed with water and purified by recrystallization from petroleum ether to give product **3** as a brown powder, with a 73% yield; m. p: 140–143 °C; IR (KBr): ν (cm^−1^) 1156 SO2, 1628 C=N, 2198 CN, 2933CH-Al, 3025CH-Ar, 3340, 3214 NH2; ^1^H-NMR (300 MHz, DMSO-d6): δ1.61–2.49 (8H, m), 5.35(2H,s,br), 7.27–7.90(14H, m), 8.40(1H,s,br); ^13^C-NMR (100 MHz, DMSO-d6): δ22.51, 25.32, 27.45, 112.70, 118.02, 122.00, 125.91, 126.66, 127.62, 128.26, 128.57, 128.70, 129.34, 138.05, 142.11, 154.35, 118.02, 161.50, 164.05; analysis (calc., found for C_32_H_29_N_7_ O_2_ S_2_): C(63.24, 63.05), H(4.81, 4.69), N(16.13, 16.00), S(10.55, 10.34).

#### Synthesis of 5-amino-2-(3-phenyl-1,3,4,5,6,7-hexahydro-2H-indazol-2-yl)furo[2,3-d] thiazole-6-carbonitrile (4)

A mixture of derivative **1** (10 mmol, 3 gm) and malononitrile (10 mmol, 0.7 gm) in dioxane (30 mL) containing a catalytic amount of triethylamine (1 mL) and/or in ethanol (25 mL) containing a catalytic amount of piperidine (0.5 mL) was heated under reflux for 5 h, cooled to RT, and acidified by HCl. The solid precipitate was filtered off, washed with distal water (3 times) and recrystallized from dioxane to give compound **4** as a brown powder in a 52% yield; m. p: 130–133 °C; IR (KBr): ν (cm^−1^) 1553C=C, 1627C=N, 2191 CN, 2935CH-Al, 3058CH-Ar, 3333, 3201NH2; ^1^H-NMR (300 MHz, DMSO-d6): δ1.66–2.49, 4.09(2H,s,br), 6.87 (2H,s,br) exchangeable with D_2_O, 7.20–7.74(5H,m), 8.15(1H,s); ^13^C-NMR (100 MHz, DMSO-d6): δ21.25, 22.04, 25.84, 26.60, 98, 108, 115.53, 120.60, 126.82, 127.40, 128.23, 129.01, 129.40, 130.18, 142.93, 162.05; analysis (calc., found for C_19_H_17_N_5_ OS): C(62.79, 62.65), H(4.71, 4.61), N(19.27, 19.03), S(8.82, 8.72).

#### Synthesis of 5-amino-2-(3-phenyl-1,3,4,5,6,7-hexahydro-2H-indazol-2-yl)thieno[3,2-d]thiazole-6-carbonitrile (5)

The ethanolic solution of derivative **1** (10 mmol, 3 gm) containing a catalytic amount of piperidine (0.5 mL), malononitrile (10 mmol, 0.7 gm), and Sulphur element (10 mmol, 0.3 gm) was heated under reflux for 4 h, filtered off on hot to get rid of an excess of Sulphur, cooled to RT, and acidified by HCl. The separated solid was filtered off, washed with water, and purified by recrystallization from toluene to give derivative **5** as a brownish-red powder, in a 69% yield; m. p: 135–137 °C; IR (KBr): ν (cm^−1^) 1561 C = C, 1621C=N, 2190 CN, 2857, 2935CH-Al, 3025, 3057CH-Ar, 3324, 3193NH_2_; ^1^H-NMR (300 MHz, DMSO-d6): δ1.56–2.49 (8H, m); 4.25(1H,s,br), 6.95(2H,s,br) exchangeable with D_2_O, 7.12–7.97 (5H, m), 8.45 (1H, s). ^13^C-NMR (100 MHz, DMSO-d6): δ 22.18, 23.65, 24.65, 26.33, 112.55, 115.00, 118.00, 125.23, 127.57, 128.20, 129.52, 143.05, 144.32, 161.55, 171.60; analysis (calc., found for C_19_H_17_N_5_ S_2_): C(60.13, 60.00), H(4.52, 4.42), N(18.45, 18.39), S(16.90, 16.69).

#### Synthesis of 5-amino-2-(3-phenyl-1,3,4,5,6,7-hexahydro-2H-indazol-2-yl)thieno[3,2-d] thiazole-6-carbohydrazide (6)

A mixture of compound **1** (10 mmol, 3gm), cyanoacetohydrazide (10 mmol, 1gm), and Sulphur element (10 mmol, 0.3 gm) in dimethylformamide (20 mL) containing a catalytic amount of piperidine (0.5 mL), was heated under reflux for 12 h, filtered off at hot to get rid of an excess of Sulphur, cooled to RT, and acidified by HCl. The solid formed was filtered off, washed with water, and purified by recrystallization from petroleum ether to give product **6** as a brownish-red powder, in a 52% yield; m. p: 160–163 °C; IR (KBr): ν (cm^−1^) 1493 C=C, 1621 C=N, 2858, 2932 CH-Al, 3025, 3058CH-Ar, 3.350, 3195NH2; ^1^H-NMR (300 MHz, DMSO-d6): δ1.64–2.33(8H,m), 4.30(2H,s,br) exchangeable with D_2_O, 4.55(1H,s,CH), 7.15–7.50(5H,m), 7.80(2H,s) exchangeable with D_2_O, 9.75(1H,s) exchangeable with D_2_O; analysis (calc., found for C_19_H_20_N_6_ OS_2_): C(55.32, 55.27), H(4.89, 4.80), N(20.37, 20.29), S(15.54, 15.49).

#### Synthesis of 7-imino-2-(3-phenyl-1,3,4,5,6,7-hexahydro-2H-indazol-2-yl)-6,7-dihydro-5H-pyrano [2,3-d]thiazol-5-one (7)

A mixture of derivative **1** (10 mmol, 3 gm) and ethyl cyanoacetate (10 mmol, 1.2 gm) in dioxane (30 mL) containing a catalytic amount of piperidine (0.5 mL) was heated under reflux for 5 h, cooled to RT, and acidified by HCl. The separated solid was filtered off, washed with water and purified by recrystallization from benzene to give the required product **7** as a brown powder, with a 37% yield; m. p: 95–98 °C; IR (KBr): ν (cm^−1^) 1547C=C, 1626C=N, 1721C=O, δ-lactone, 2935 CH-Al, 3025CH-Ar, 3345 NH. ^1^H-NMR (300 MHz, DMSO-d6): δ1.67–2.49(8H,m); 3.88(2H,s,br); 7.22–7.52(5H,m); 8.15, 8.40 (2H,s) with D_2_O exchangeable.^13^C-NMR(100 MHz, DMSO-d6): δ*21.85*, 23.59, 24.45, 26.33, 62, 94.40, 120.49, 12517, 125.66, 126.76, 127.19, 128.24, 129.30, 130.91, 131.98, 133.39, 135.77, 146.34, 151.44, 155.09, 156.20; analysis (calc., found for C_19_H_18_N_4_ O_2_S): C(62.28, 62.11), H(4.95, 4.89), N(15.29, 15.18), S(8.75, 8.68).

#### Synthesis of 2-(1-amino-5-(3-phenyl-1,3,4,5,6,7-hexahydro-2H-indazol-2-yl)-1H-pyrazolo [3,4-d] thiazol-3-yl)acetonitrile (8)

An ethanolic solution of derivative **1** (10 mmol, 3 gm) containing a catalytic amount of piperidine (0.5 mL) and cyanoacetohydrazide (10 mmol, 1 gm) was heated under reflux for 12 h, cooled to RT, and acidified by HCl. The solid formed was filtered off, washed with water, and purified by recrystallization from toluene to give product **8** as a pale brown powder, in a 70% yield; m. p: 170–173 °C; IR (KBr): ν (cm^−1^) 1493C=C, 1628C=N, 2213CN, 2857, 2933CH-Al, 3025, 3057CH-Ar, 3191NH; ^1^H-NMR (300 MHz, DMSO-d6): δ1.59–2.97(8H,m), 3.39(2H,s,br), 7.27–7.86(5H,m), 10.63(1H,s); analysis (calc., found for C_19_H_18_N_6_S): C(62.96, 62.92), H(5.01, 4.89), N(23.19, 23.13), S(8.85, 8.78).

#### Synthesis of ethyl 2-(3-phenyl-1,3,4,5,6,7-hexahydro-2H-indazol-2-yl)-6-thioxo-6,6a-dihydrothieno [3,4-d]thiazole-4-carboxylate (9)

A mixture of compound **1** (10 mmol, 3 gm), ethyl chloroacetate (10 mmol, 1.2 gm), and carbon disulfide (10 mmol, 0.8 gm) in dry dioxane (30 mL) containing anhydrous K_2_CO_3_ (1.4 gm) was stirred at RT for 12 h, and filtered off to remove excess K_2_CO_3_. The filtrate was poured onto ice water. The precipitate was filtered out, washed three times with deionized water and purified by recrystallization from benzene to give the required product **9** (orange powder) in a 32% yield; m. p: 85–88 °C; IR(KBr): ν(cm^−1^)1028C-S, 1205C=S, 1583C=C, 1621C=N, 1731C=O, 2858, 2932CH-Al, 3025, 3057CH-Ar, 3411NH. ^1^H-NMR (300 MHz, DMSO-d6): δ1.18(3H,t); 1.62–2.31(8H,m); 3.99(1H,s); 4.12 (2H,q); 4.50(1H,s); 7.12–7.86 (5H,m); 8.55(1H,s); analysis (calc., found for C_21_H_21_N_3_ O_2_ S_3_): C(56.86, 56.76), H(4.77, 4.70), N(9.47, 9.38), S(21.68, 21.58).

### Evaluating the biological activities of the newly synthesized compounds

#### Cytotoxicity and antitumor activity evaluation

The human melanocytes (HFB-4) cells, hepatoma (HepG-2) cells, and colon carcinoma (Caco-2) cells were obtained from American Type Culture Collection (ATCC, USA). The cytotoxicity of the newly synthesized compounds (at various concentrations) was determined using the 3-[4, 5-dimethylthiazol]-2, 5-diphenyltetrazolium bromide method (MTT assay)^59^ against HFB-4 cells (normal human melanocytes), HepG-2 cells (hepatoma), and Caco-2 cells (colon carcinoma). Normal and cancer cells (1.0 × 10^4^) were cultured in sterile 96-well microplates, and incubated for 24 h in DMEM (Lonza, USA) supplemented with 10% fetal bovine serum (Gibco, USA) for HFB-4 and Caco-2 cells or in an RPMI-1640 medium (Lonza, USA) supplemented with 10% FBS for HepG-2 cells. After 24 h of incubation, different doses of the tested synthesized compounds (2–64 g/mL) were added in triplicate to all cells and incubated for another 48 h in 5% CO_2_. After washing the cells 3 times with fresh media, to remove dead cells and debris, a solution of MTT (Sigma -Aldrich, 0.5 mg/mL) was added to each well and further incubated for 2–5 h at 37 °C. Afterward, the MTT solution was decanted and 200 µL of DMSO was added. The optical density was measured at 570 nm using a microplate reader (BMG LabTech, Germany), where the cells’ relative viability (%) was estimated using the following equation: viability of cells (%) = [A_1_ − A_0_/A_U_ − A_0_] × 100, where A1 is the absorbance of the test compound, A_0_ is the absorbance of the blank, and A_U_ is the absorbance of untreated cells (control).

The antitumor activity of the synthesized compound was determined by calculating the IC_50_ value (half maximal inhibitory concentration) using Graph Pad Prism 6.0 software. The value of IC_50_ indicates the derivative concentration that causes 50% cell death, whereas the value of the selectivity index (SI), indicating the ratio of the IC_50_ value of normal cells versus the IC_50_ value of tumor cells, was also included as reported by^60,61^. Furthermore, the effect of the highly active antitumor derivatives (**4**, **6**, and **8**) on the morphology of HepG-2 and Caco-2 cells was explored at different concentrations (4–16 μg/mL) by using phase-contrast microscopy (Olympus, Germany) and compared to untreated cells (negative control).

#### The influence of the newly synthesized derivatives on the expression level of some tumor regulating genes

The effects of the potent antitumor derivatives (**4**,** 6**, and **8**) on the expression level of some tumor regulating genes were elucidated through quantitative real-time PCR (qRT- PCR) and compared to 5-fluorouracil (5-FU) as a reference antitumor drug. Five genes were assessed, including the tumor oncogene (Bcl-2), tumor suppressor gene (p53), matrix metalloproteinase gene (MMP-9), vascular endothelial growth factor gene (VEGF), and beta-catenin protein gene (β-catenin), in HepG-2 and Caco-2 cells, before and after treatment for 2 days with IC_50_ concentrations for compounds **4**, **6**, and **8**. Following the extraction of total RNA from the tested cells, using the Gene JET RNA Purification Kit (Thermo Scientific, USA), cDNA synthesis was performed according to the cDNA Synthesis Kit protocol (Thermo Scientific, USA). qRT PCR was performed using the SYBR green kit and specific primers (Forward/Reverse) as follows: 5′-TCCGATCAGGAAGGCTAGAGTT-3'/5'-TCG GTCTCCTAAAAGCAGGC-3′ for the Bcl-2 gene, 5′-TAACAGTTCCTGCATG GG CG GC-3′/5′-AGGACAGGCACAAACACGCACC-3′ for p53, 5'-CTGCGTATTTCCATT CATC-3'/5'-CCTTGGGTCAGGTTTAGAG-3' for the MMP-9 gene, 5'-GGCTTTACT GCTGTACCTCC-3'/5'-CAAATGCTTTCTCCGCTCT-3' for the VEGF gene, and 5'-CATATGCGGCTGCTG TTCTA-3'/5'-CCGAAAG CCGTTTCTTGTAG-3' for the β-catenin gene. The upregulation and/or downregulation of the expression of the tested genes in Caco-2 and HepG-2 cells was determined by using the equation  2^−ΔΔCT^^60^.

#### Cell cycle arrest analysis

The cell cycle arrest of treated Caco-2 cells was evaluated through flow cytometry (Partec, Germany) in comparison with untreated cells and treated cells with 5-FU as a positive control^60,62^. After treatment of Caco-2 cells with the most potent compounds at IC_50_ concentrations, the cells were resuspended in 1 mL of cold PBS, pH, 7.2. After washing the cells three times, the cells were fixed by adding 1 mL of 70% cold ethanol dropwise with a gentle vortex. Caco-2 cells were washed three times again with cold PBS and incubated in 1 mL of PBS containing 5 μg/mL RNase A (Sigma-Aldrich) for 1 h at 37 °C. Then 10 μL of PI (Sigma-Aldrich) was added to cells at a final concentration of 1 mg/mL in deionized water and left at 4 °C until analysis in the dark. The cell cycle of Caco-2 cells before and after treatment was analyzed by FACS using Cell Quist and Mod Fit software by reading at 488 nm.

#### Molecular docking analysis

The binding orientations and interactions of the potent antitumor derivatives (**4**, **6**, and **8**) into five tumor-regulating proteins, namely MMP-9, p53, β-catenin, Bcl-2, and VEGF, were simulated using Molecular Operating Environment (MOE) 2014) software. The three-dimensional structure (3D) of the selected proteins was downloaded from the PDB website. The water molecules and repeated chains were removed. Protons were added and the energy of the protein was minimized. The isolation of the pocket was then carried out. Validation of the downloaded structure was confirmed by re-docking the downloaded ligand into the isolated pocket. The obtained root mean square deviation (RMSD) was found to be lower than 1.5 Å. The preparation of potent antitumor derivatives (**4**, **6**, and **8**) for docking was carried out by the construction of chemical structures at the MOE. Protons were then added to the 3D structure. Finally, the energy was minimized using Force Field MMFF94x. The prepared structures were added to the created database. MOE conducted the docking of the newly synthesized compounds, calculated the binding energies, and provided their binding modes of them^11^.

#### Evaluation of the antimicrobial activity of the prepared compounds

The antimicrobial activity of the newly synthesized compounds **(2–9)** was evaluated against three model pathogenic microorganisms as follows: *Streptococcus mutans* ATCC 25175 (Gram-positive bacterium); *Pseudomonas aeruginosa* ATCC 27853 (Gram-negative bacterium); and *Candida albicans* ATCC 10231 (fungi-like unicellular organism). After cultivating the three tested pathogens overnight in LB broth, 100 µL of each tested organism (10^6^ CFU/mL) was added separately to 100 µL of the serially diluted test compounds (5–80 µg) in a 96-well tissue culture plate and incubated at 37 °C for 24 h. Eventually, microbial growth was measured at 600 nm, and the results are presented in the form of MIC (Table 4). Two antibacterial reference drugs, ampicillin and ciprofloxacin, and one antifungal drug, clotrimazole, were incorporated into the experiments.

#### Evaluation of microbial-biofilm inhibition activity of the newly synthesized compounds

The activity of the newly synthesized compounds against microbial biofilm formation was estimated through the tissue-culture plate technique (TCP) as follows: the three above-mentioned pathogens, *Streptococcus mutans*, *Pseudomonas aeruginosa*, and *Candida albicans* were cultivated overnight in LB broth at 37 °C. Afterward, 200 µL of each diluted organism (10^6^ CFU/mL) was inoculated separately (in triplicate) into 96-well tissue culture plates and incubated overnight at 37 °C. The free planktonic cells were removed, after the incubation period, by flipping the plate and washing each well three times with phosphate-buffered-saline (PBS), pH 7.1. To each well, 175 µL of fresh LB broth was added along with 25 µL of the tested compound (25 µg/mL final concentrations). The plates were reincubated at 37 °C for another 24 h, washed with PBS buffer pH 7.1 three times, and dried for 5 min at 50 °C. The formed biofilm was stained with crystal violet solution (0.1% w/v) for 5 min. The excess stain was decanted, and the stained biofilm was solubilized with 200 µL of glacial acetic acid (30% v/v), where the developed blue color was measured at 590 nm. The results are expressed as relative inhibition (%) compared to untreated groups (control). Controls were prepared by culturing the three pathogens on LB medium without any tested compounds.

#### Statistical analysis

All experiments were performed in triplicate (n = 3), and all data are expressed as the mean ± SEM. The significance of statistical analysis was evaluated by the multiple comparisons of Tukey’s post-hoc test of the one-way analysis of variance (ANOVA) using the SPSS16 program, and differences were considered statistically significant at *p* values < 0.05.

### Ethical approval

This article does not contain any studies with human or animal subjects.

## Conclusion

In this study, a new series of indazolylthiazole moieties was effectively generated and described in a new range of novel pyridine, pyran, furan, thiophene, and pyrazole-carrying compounds. The newly synthesized compounds showed great potency as selective anticancer drugs against both HepG-2 and Caco-2 cell lines, with high SI values and low IC_50_ values. The antitumor activity of the synthesized derivatives included obvious tumor cell damage and stimulated a clear alteration of the cell morphology in a dose-dependent manner. Among the tested compounds, derivatives **6** and **4** revealed potent antitumor activity, where derivative **8** showed the highest antitumor activity toward both tested tumor cells with SI values of approximately 26 and IC_50_ values of 5.9 μg/mL, attributed to the presence of a thiazolylpyrazole moiety, with acetonitrile, in the pyrazole ring. The gene expression level study confirmed apoptosis induction through upregulation of the p53 gene (2–eightfold) in both treated HepG-2 and Caco-2 cells. On the other hand, compound **3** revealed significant broad-spectrum antibacterial activity against *Streptococcus mutans* (MIC of 11.2 µg/mL) and *Pseudomonas aeruginosa (*MIC of 18.29 µg/mL), comparable to that of ampicillin MIC (13.5 µg/mL) and ciprofloxacin (18.7 µg/mL), which could be attributed to the incorporated thiazolopyridine ring. The newly prepared compounds revealed low to medium antifungal activity against *Candida albicans* with a maximum antifungal activity through compound **3** (36% clotrimazole activity). Many synthesized compounds revealed antibiofilm formation activities (58.5–79% inhibition) against the three applied pathogens. Collectively, the results confirmed the effectiveness of newly synthesized compounds as promising antitumor drugs with antimicrobial activity. The current study results encourage our research team to go deeper into the exact antitumor/antimicrobial mechanisms of the newly prepared potent derivatives and explore the structural–functional relationship.

## Supplementary Information

Supplementary Information 1.

Supplementary Information 2.
