# Supplementary material for: Synthesis, and docking studies of novel heterocycles incorporating the indazolylthiazole moiety as antimicrobial and anticancer agents
Source: Sci Rep. 2022 Mar 2;12:3424. doi: 10.1038/s41598-022-07456-1 (PMC8891364; doi:10.1038/s41598-022-07456-1)
Supplement: Supplementary file 1 — Supplementary Information 1. [file 41598_2022_7456_MOESM1_ESM.docx]

 **Figure 13.** Synthesis of the new series of heterocycles-indazolylthiazole starting with an indazolylthiazolidinone precursor.
