# Supplementary material for: Synthesis, and docking studies of novel heterocycles incorporating the indazolylthiazole moiety as antimicrobial and anticancer agents
Source: Sci Rep. 2022 Mar 2;12:3424. doi: 10.1038/s41598-022-07456-1 (PMC8891364; doi:10.1038/s41598-022-07456-1)
Supplement: Supplementary file 2 — Supplementary Information 2. [file 41598_2022_7456_MOESM2_ESM.docx]

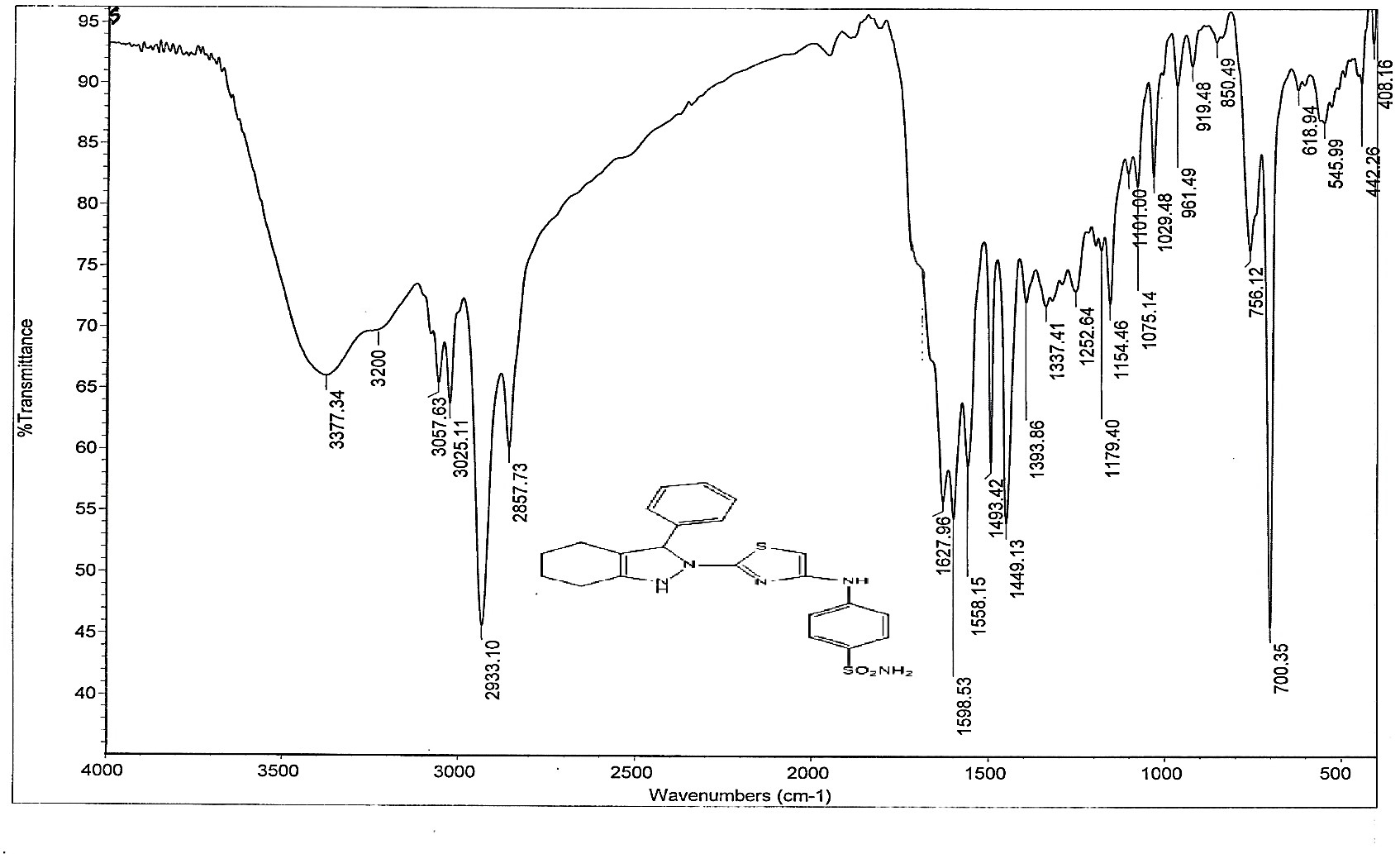

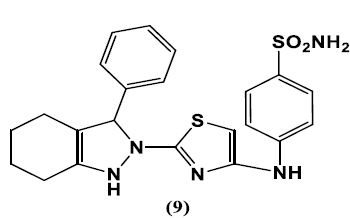


Fig. (S1 a). IR spectrum of compound 2


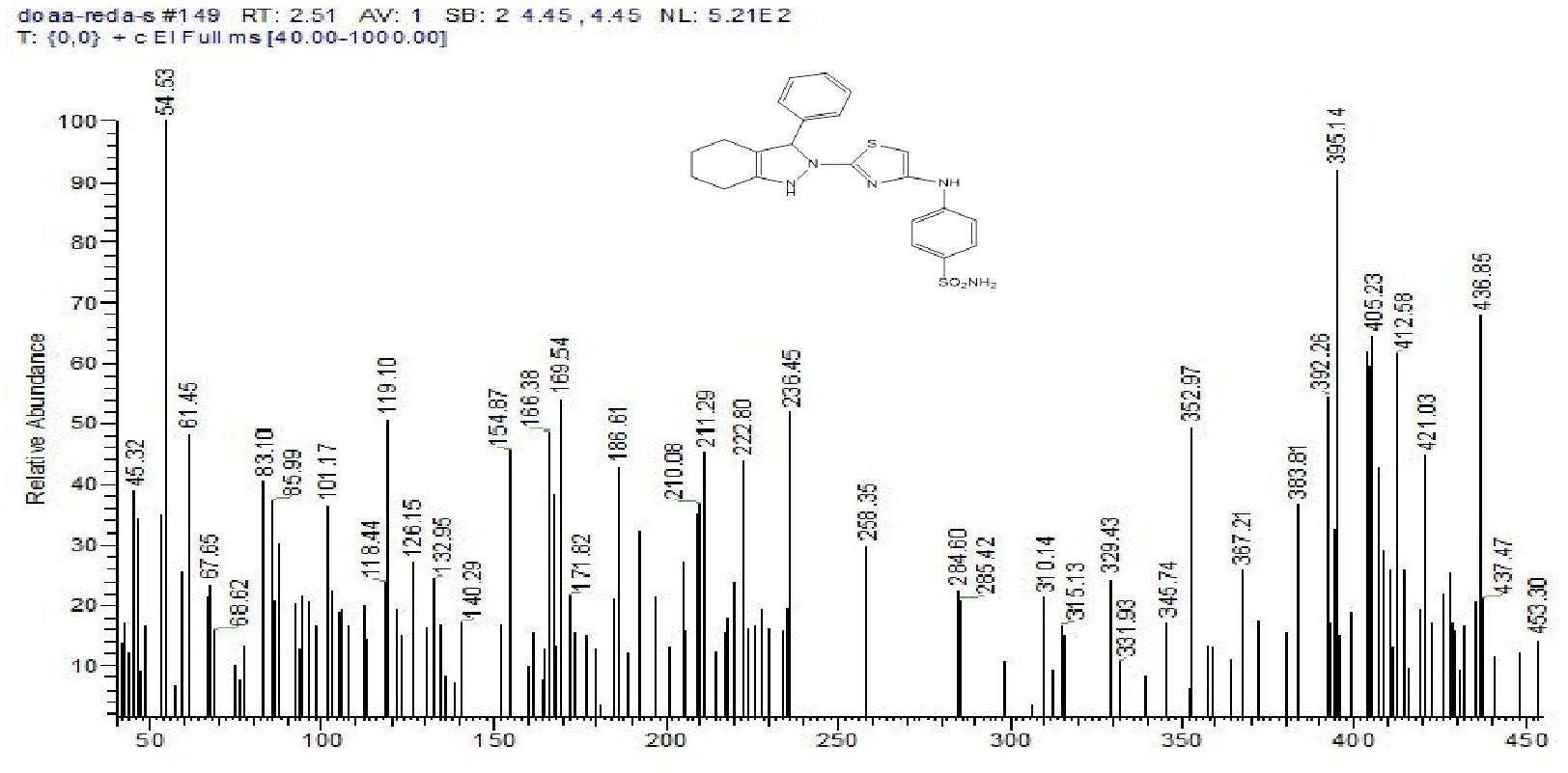

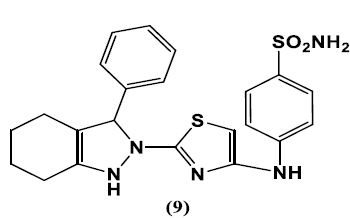


Fig. (S1 b).Mass spectroscopy of compound 2


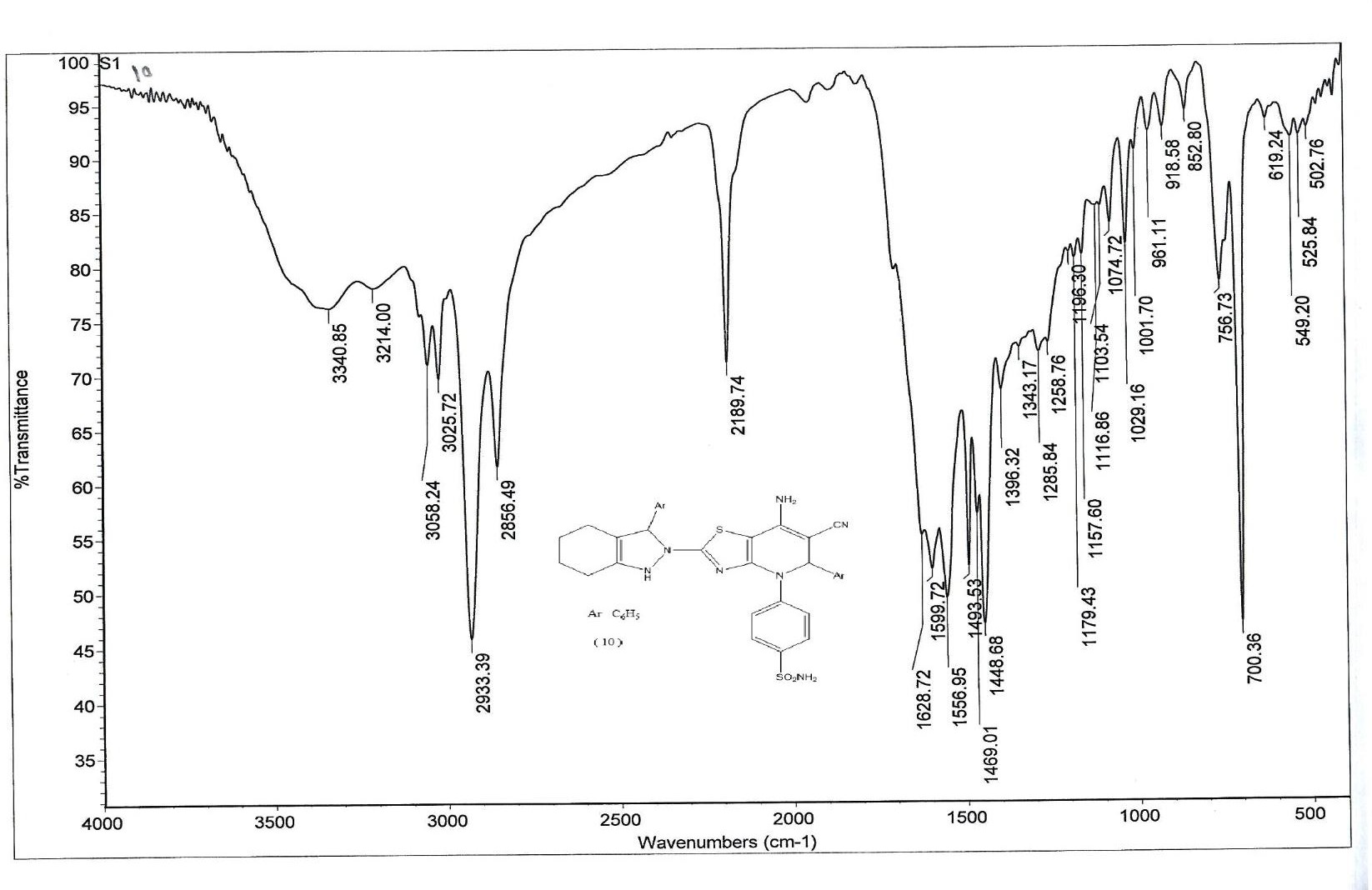

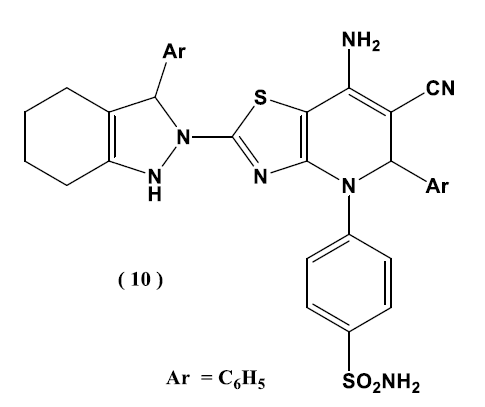


3

Fig. (S2 a).IR spectrum of compound 3


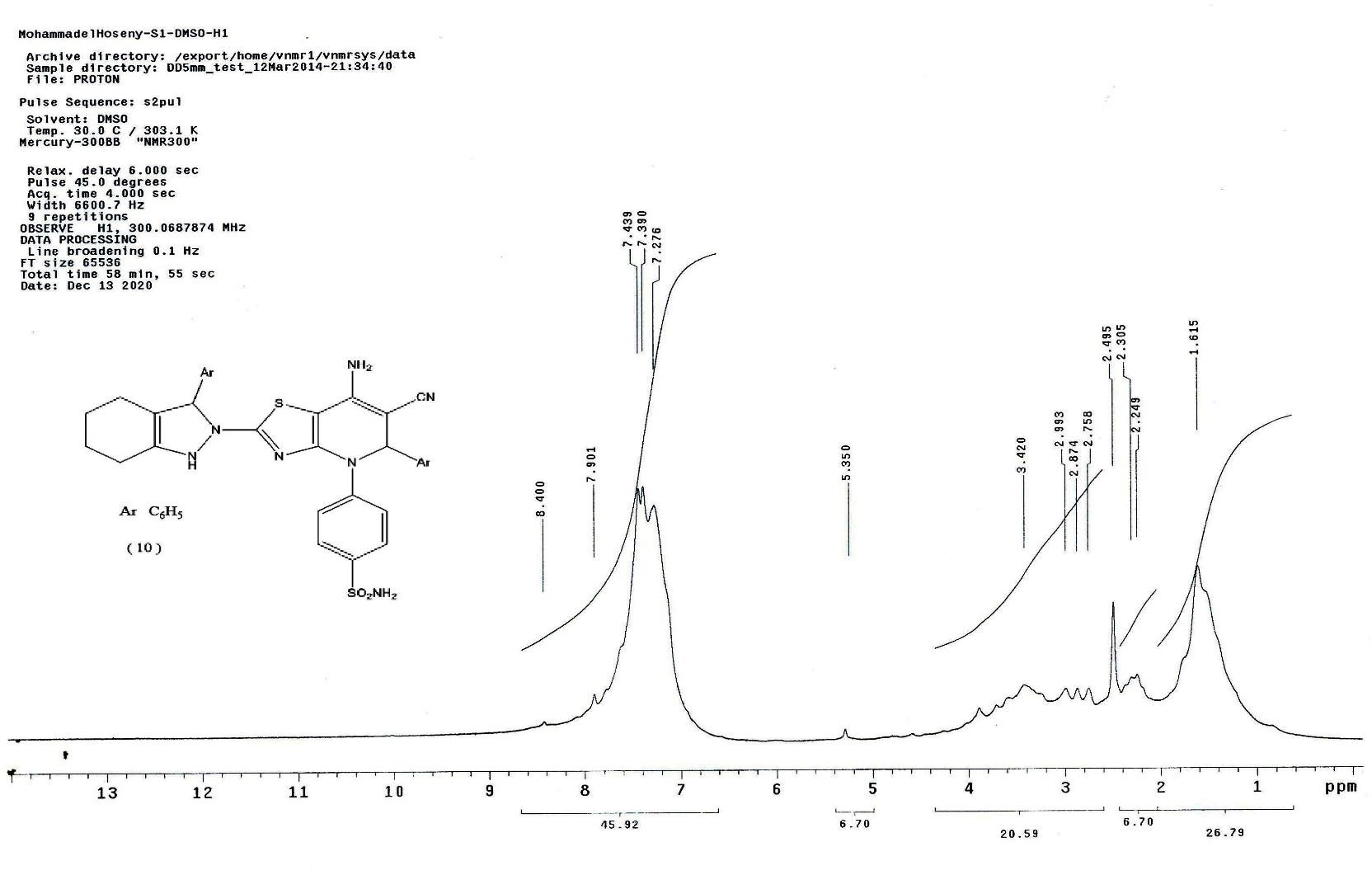

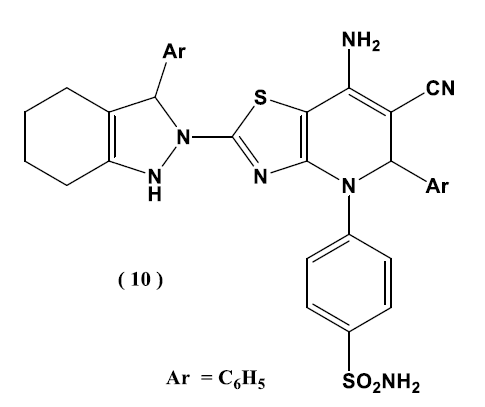


3

Fig. (S2 b). 1H-NMR spectrum of compound 3


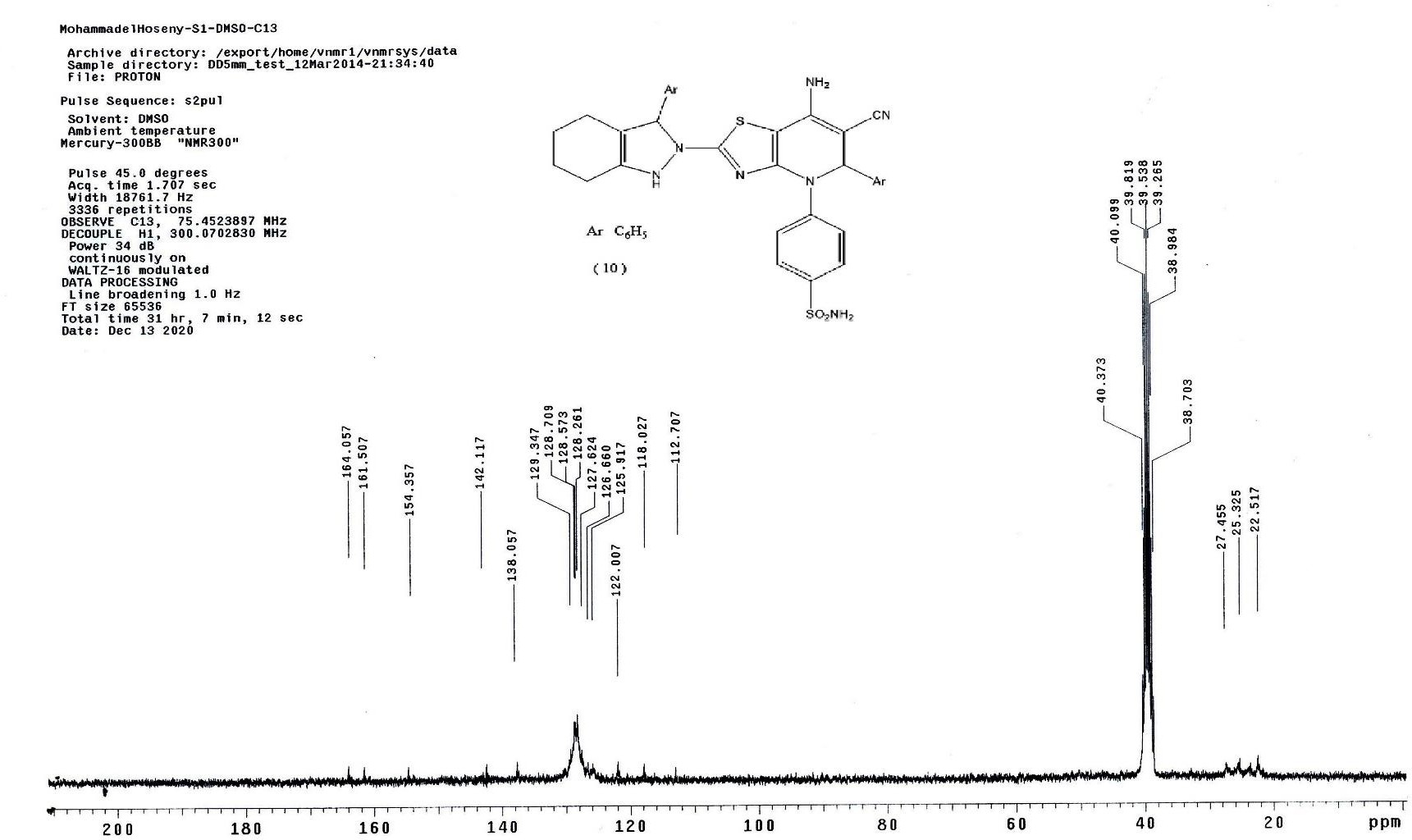

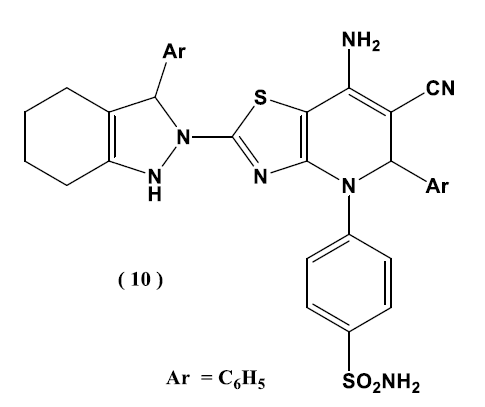


3

Fig. (S2 c). 13C-NMR spectrum of compound 3


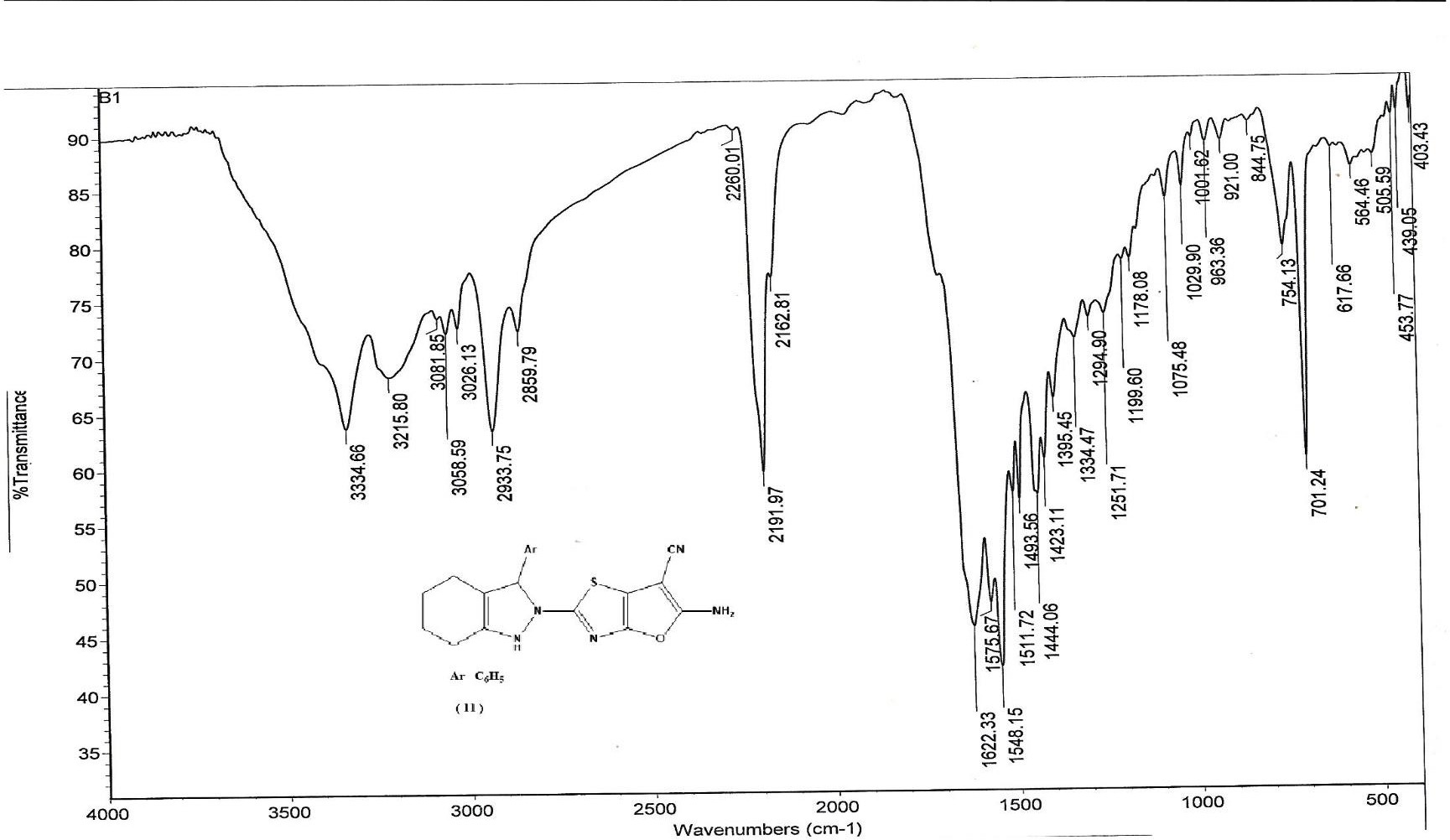

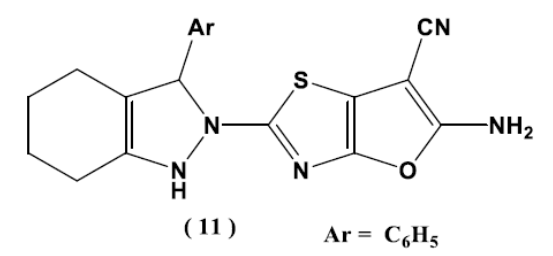


4

Fig. (S3 a). IR spectrum of compound 4


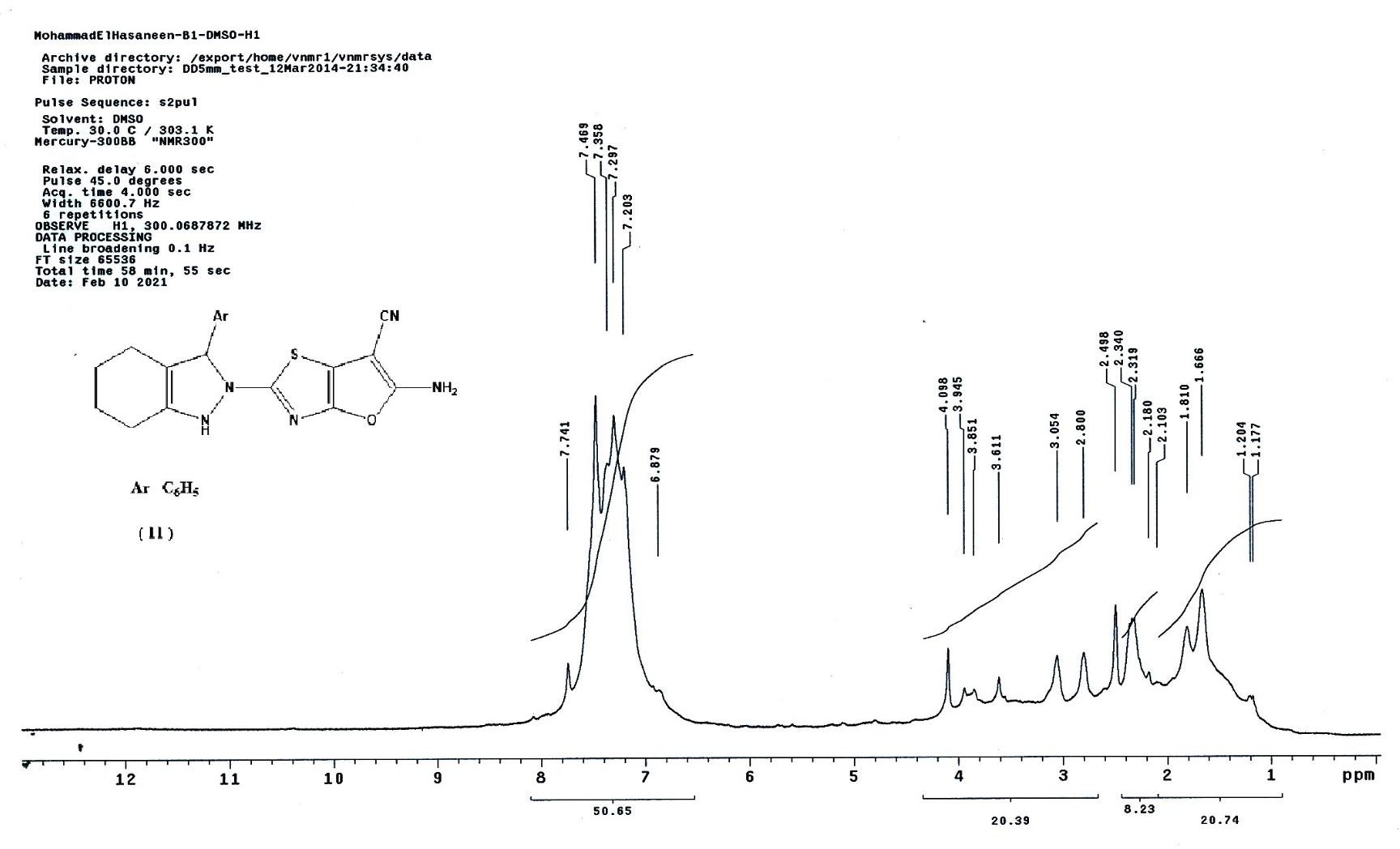

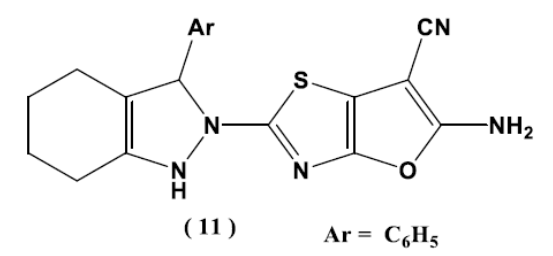


4

Fig. (S3 b). 1H-NMR spectrum of compound 4


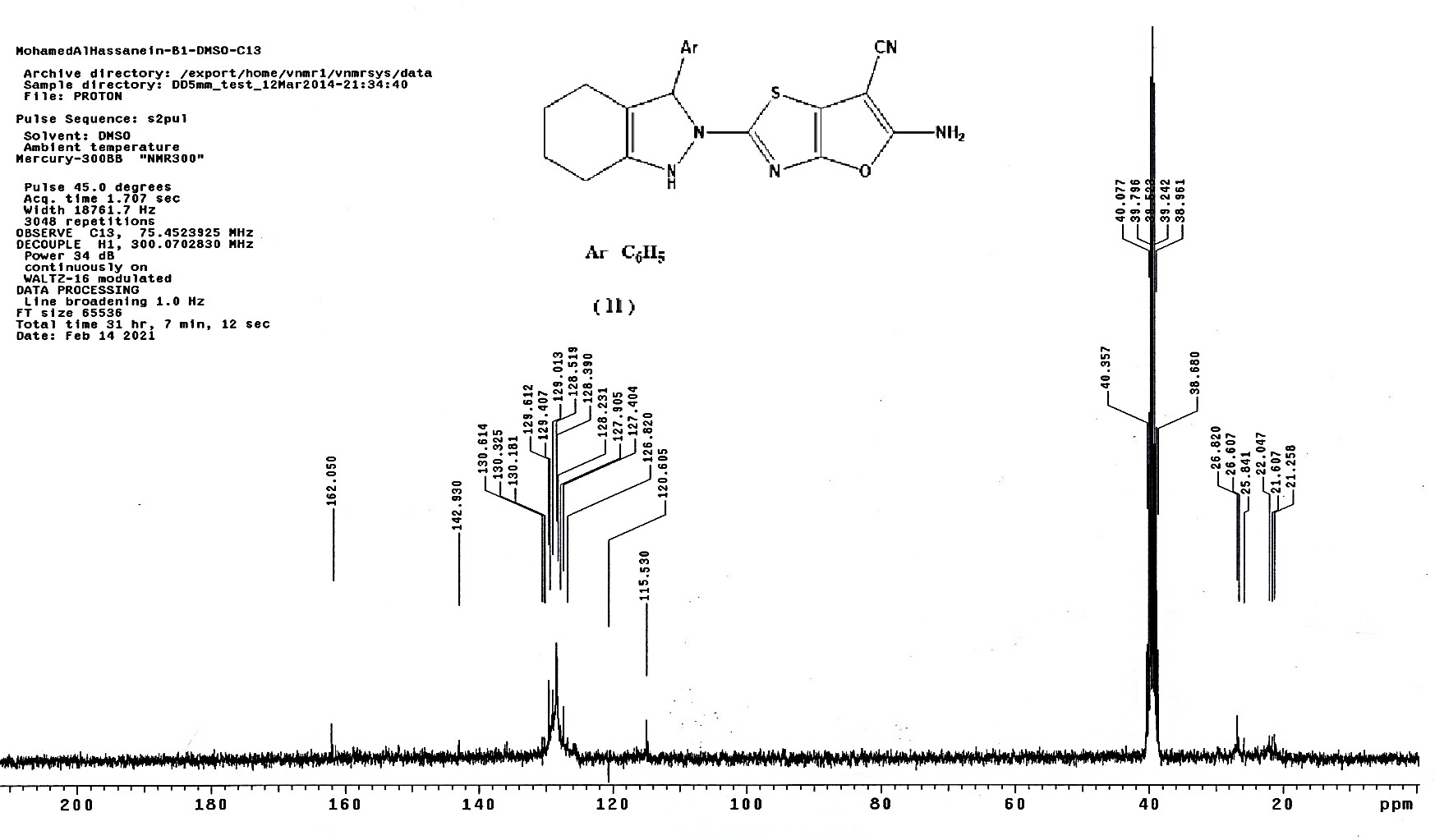

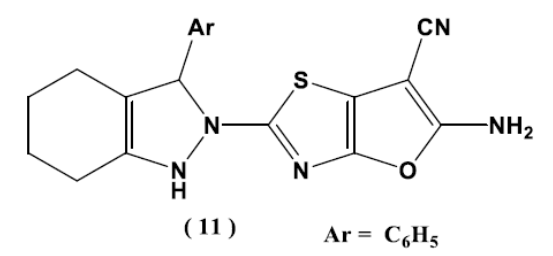


4

Fig. (S3c).13C-NMR spectrum of compound 4


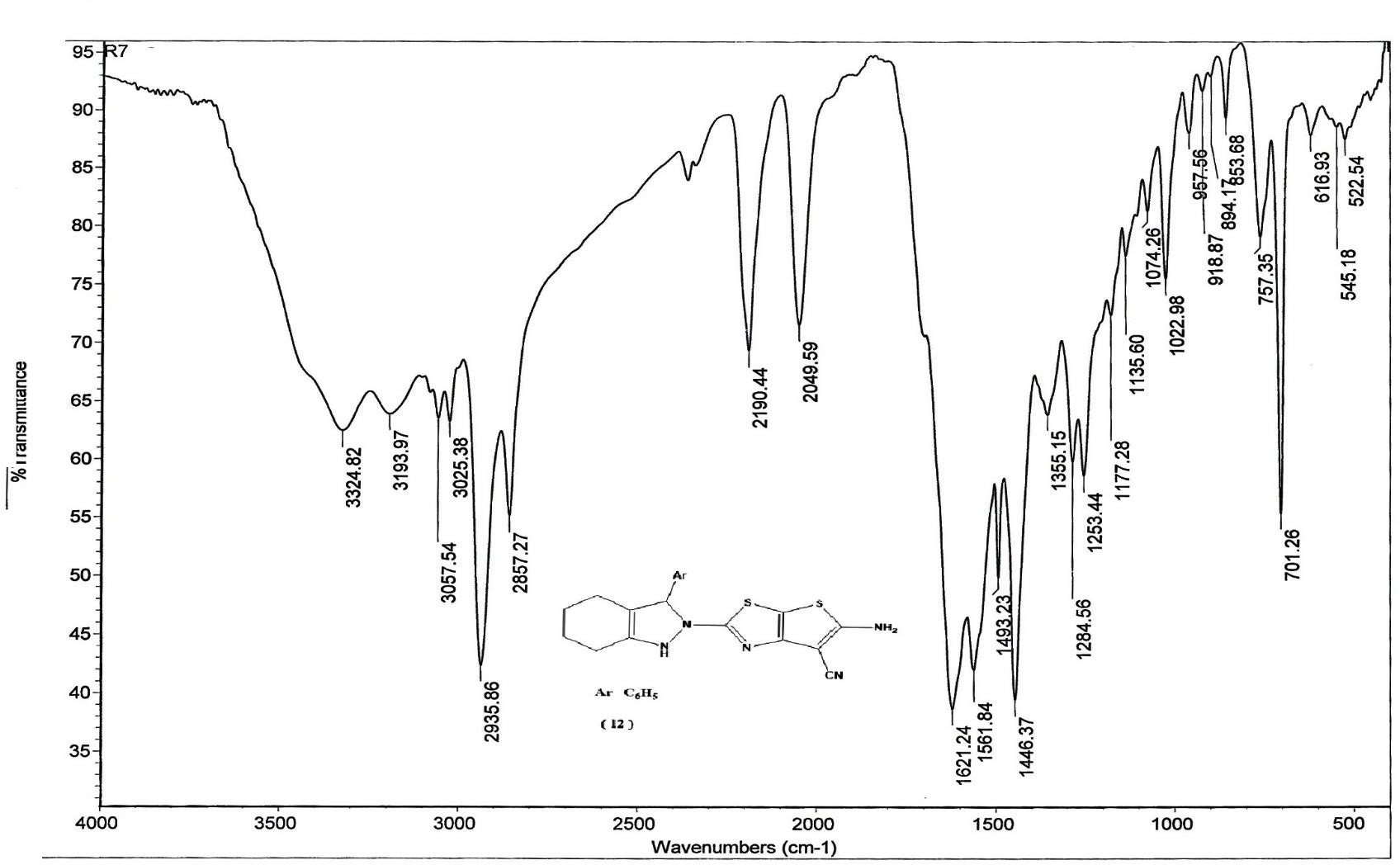

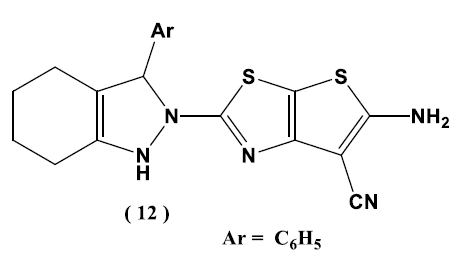


5

Fig. (S4 a). IR spectrum of compound 5


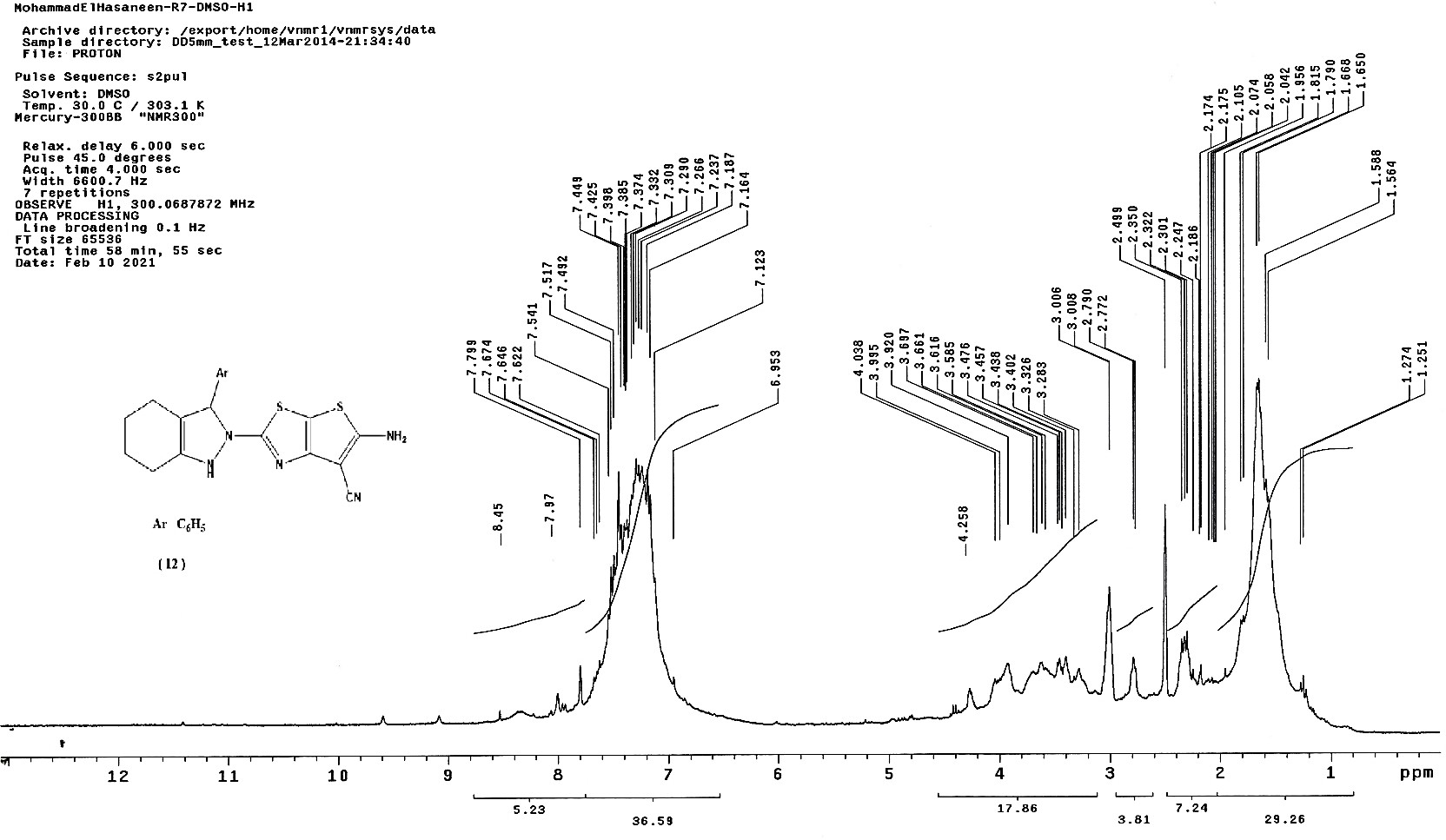

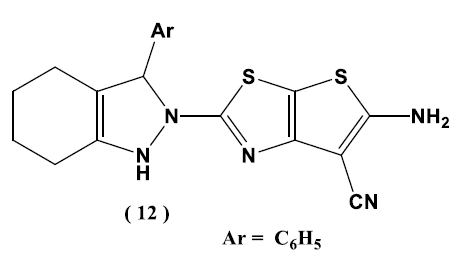


5

Fig. (S4 b). 1H-NMR spectrum of compound 5


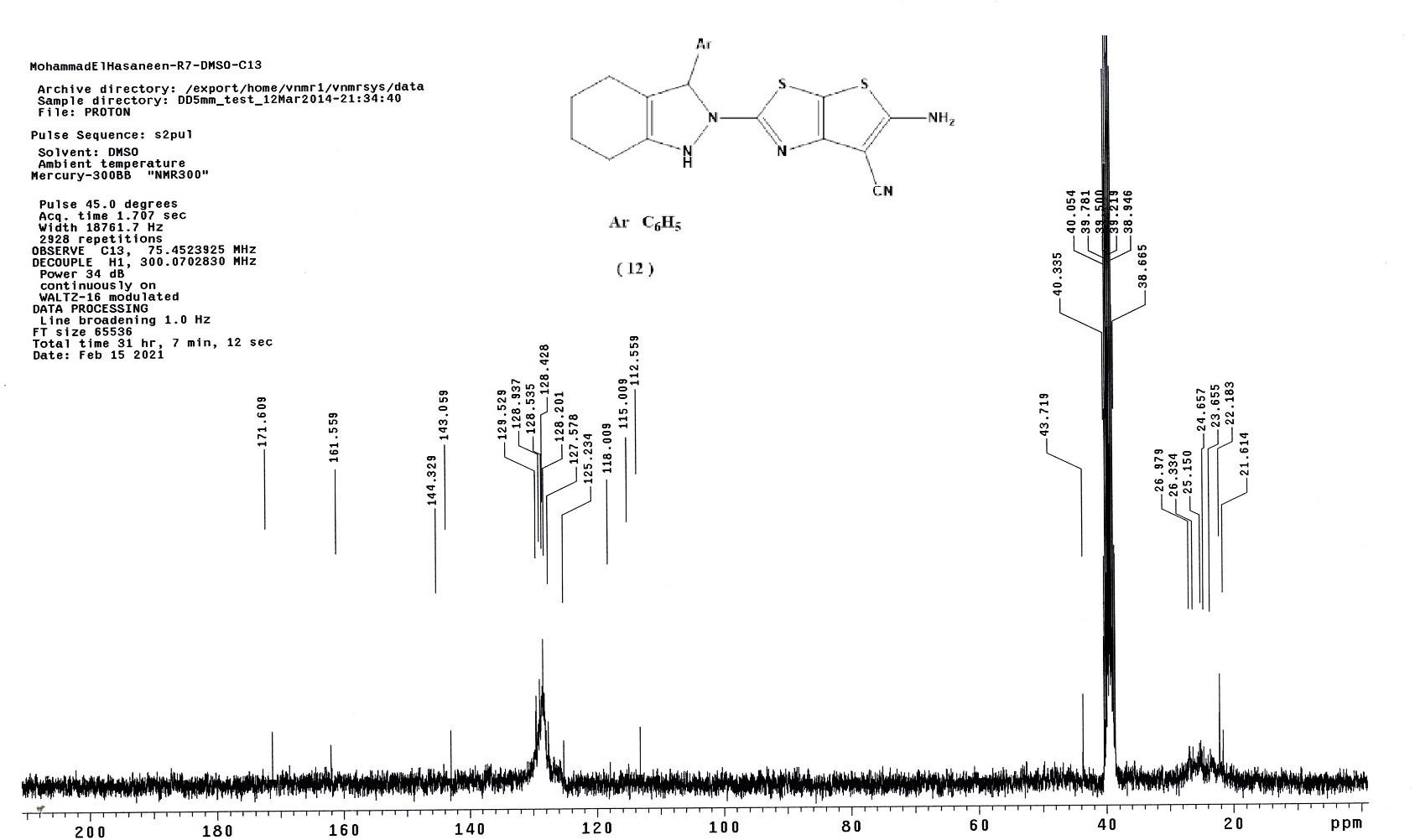

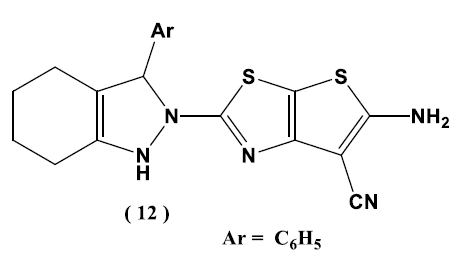


5

Fig. (S4 c). 13C-NMR spectrum of compound 5


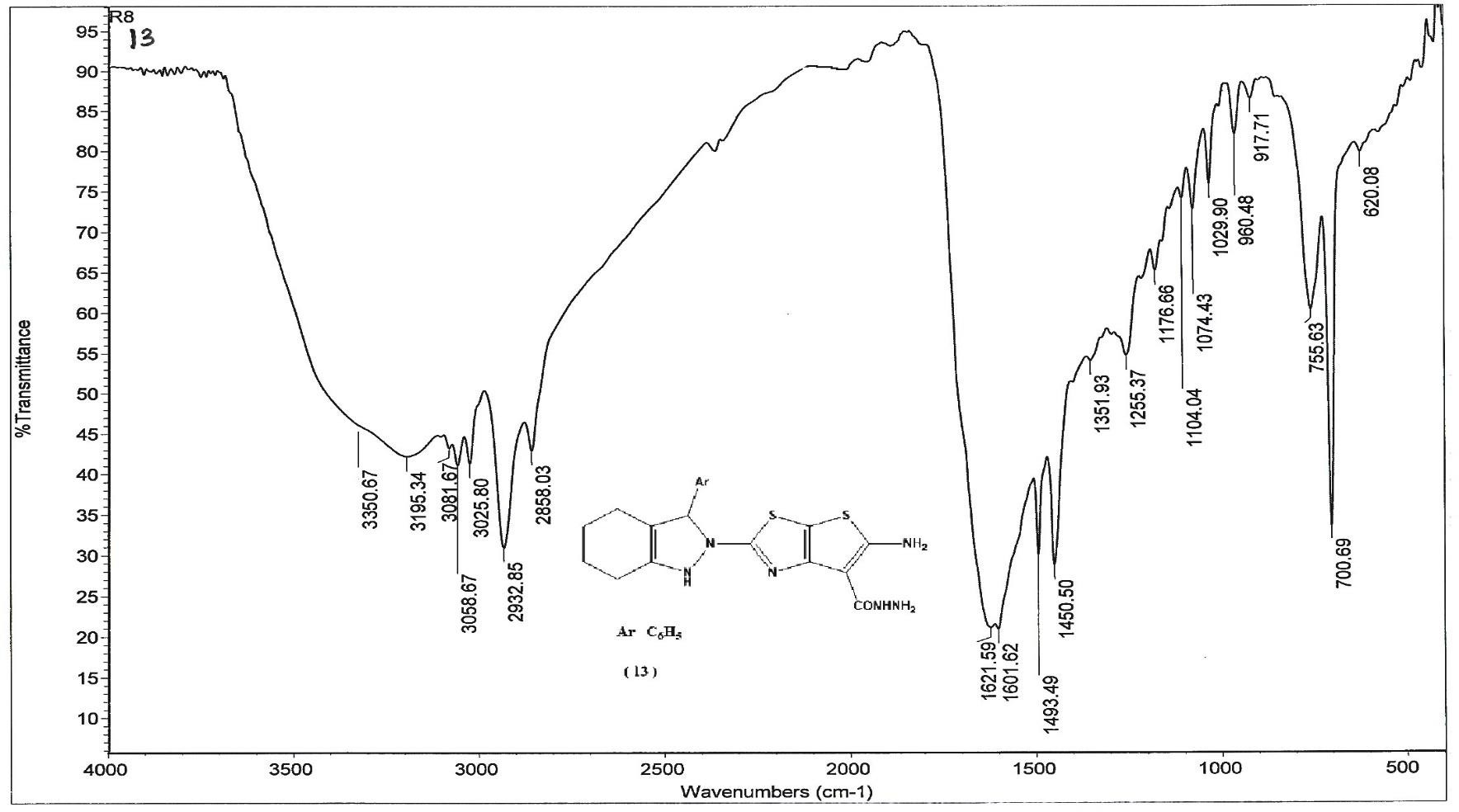

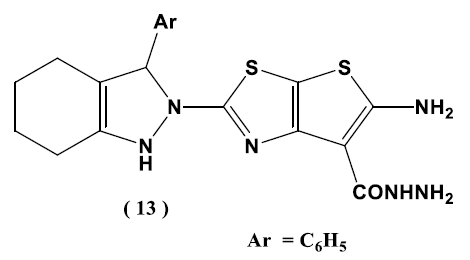


6

Fig. (S5 a).IR spectrum of compound 6


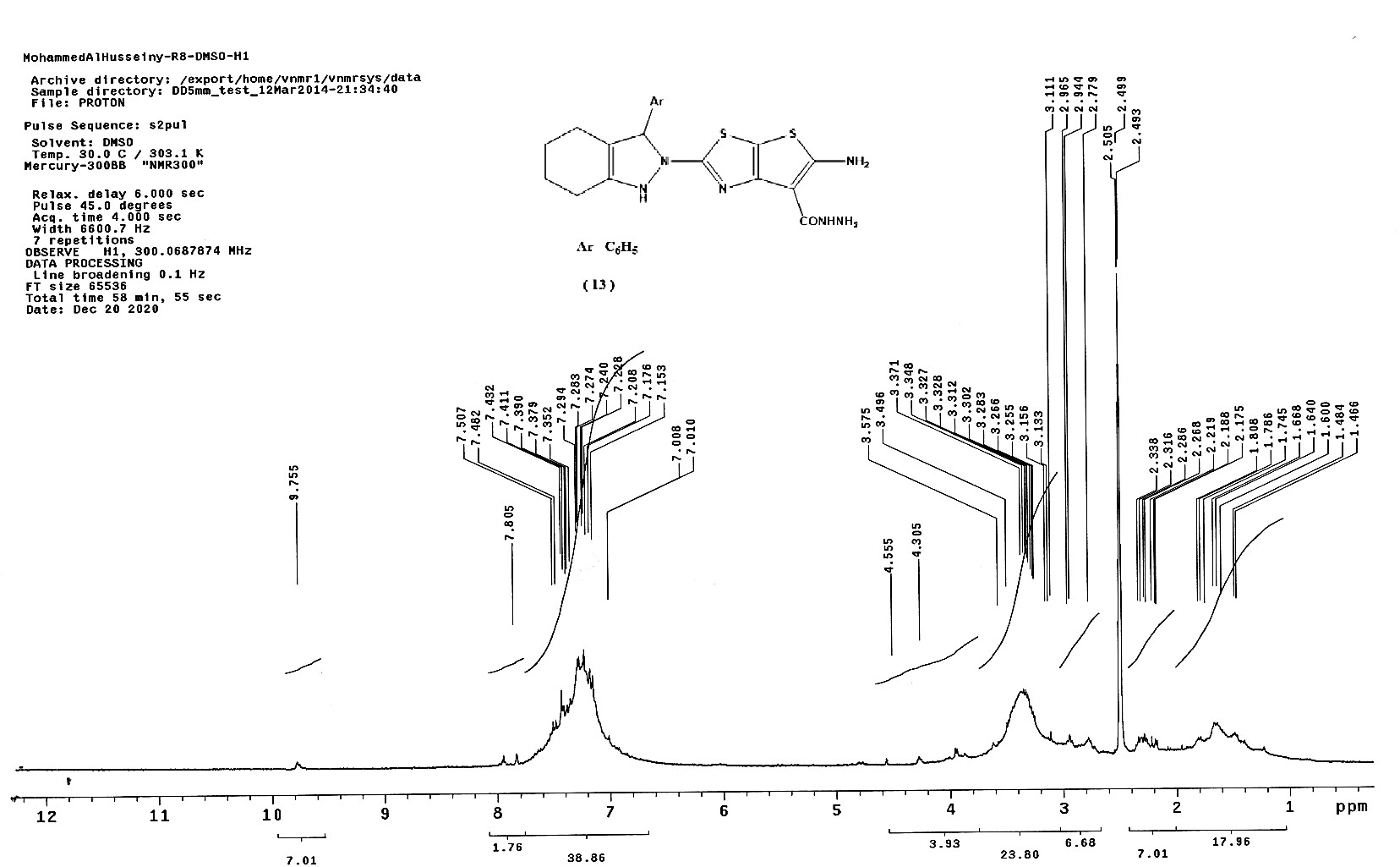

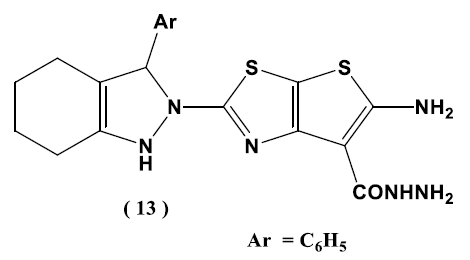


6

Fig. (S5 b). 1H-NMR spectrum of compound 6


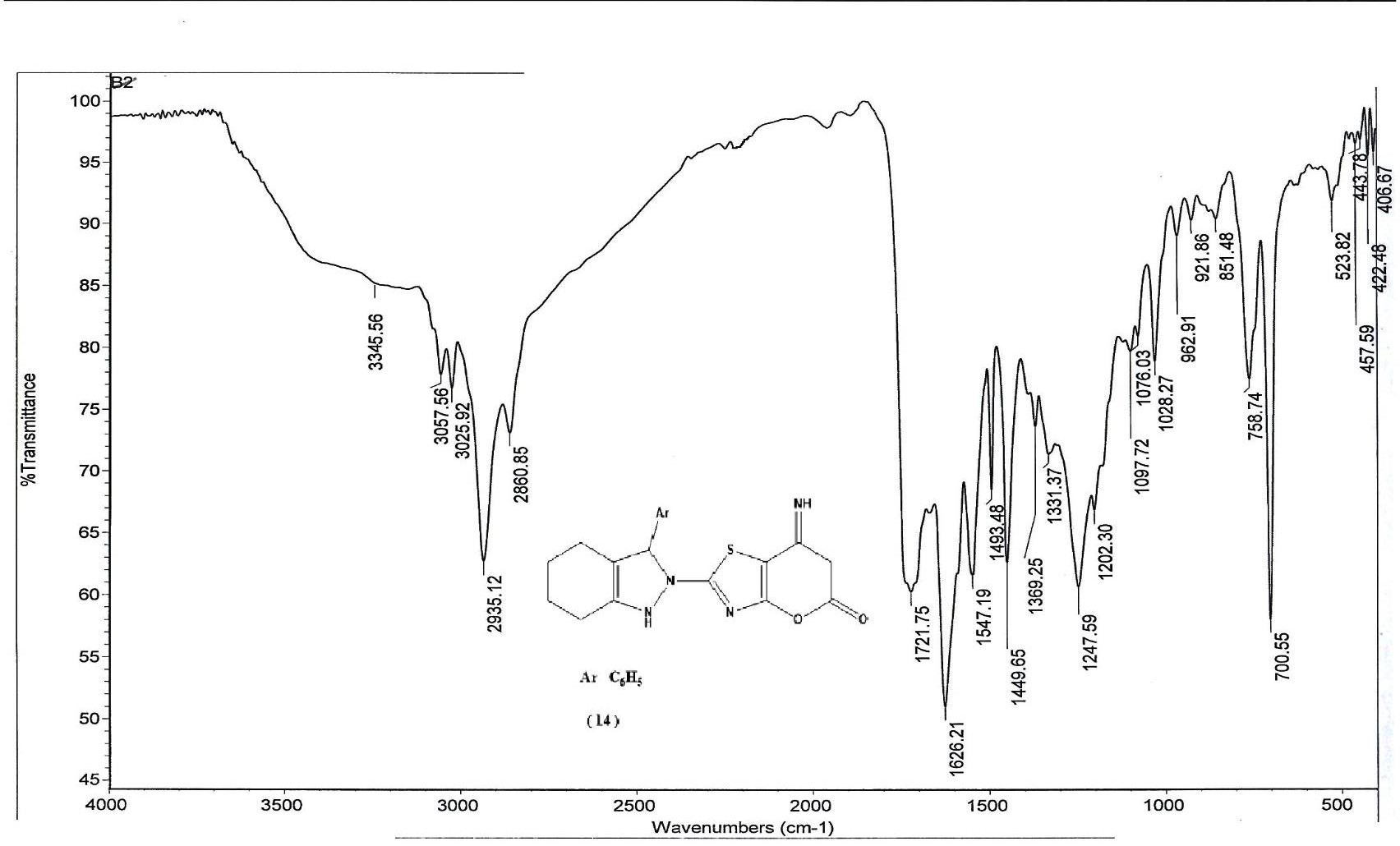

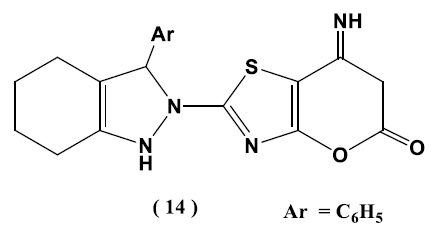


7

Fig. (S6 a). IR spectrum of compound 7


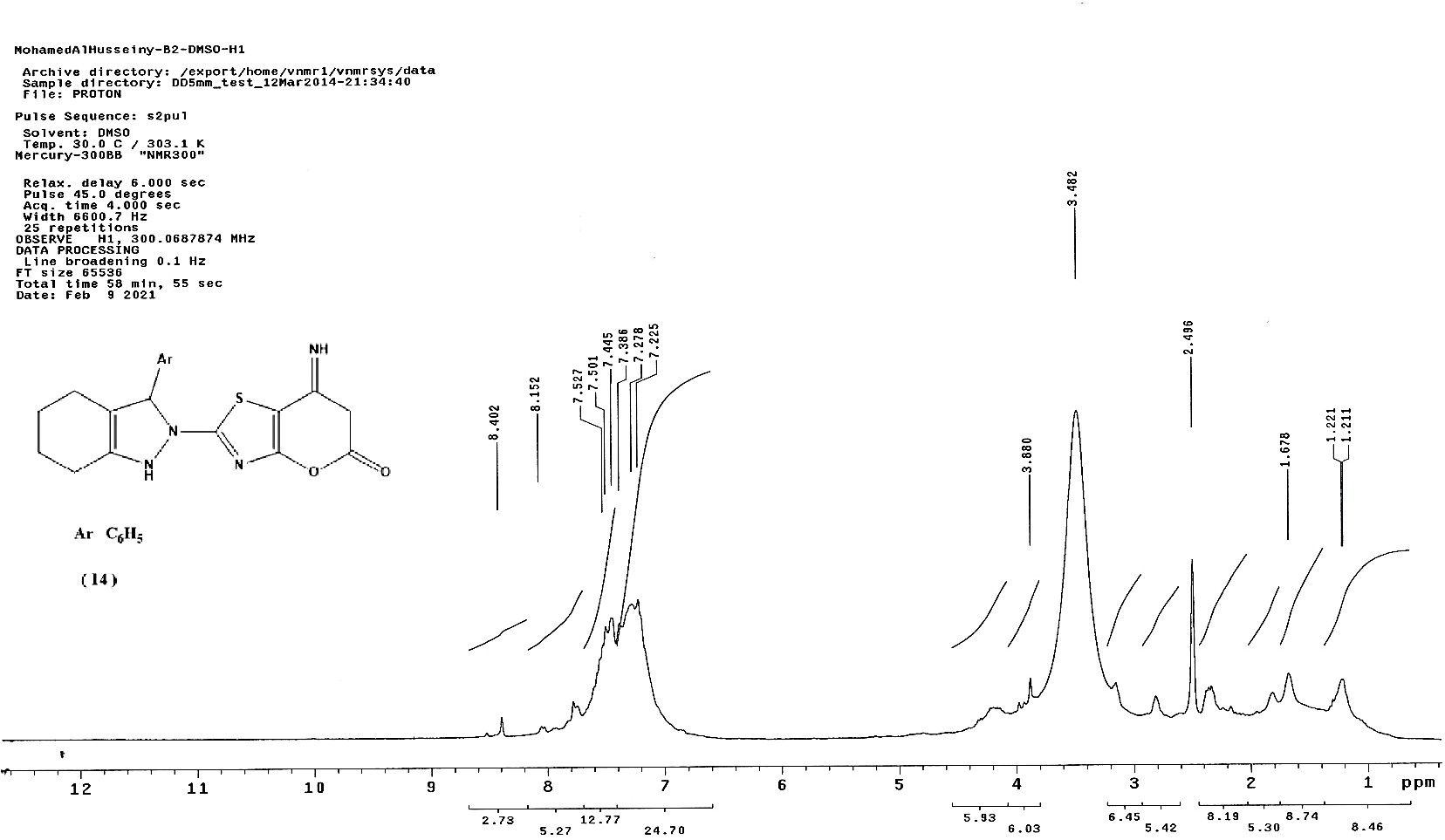

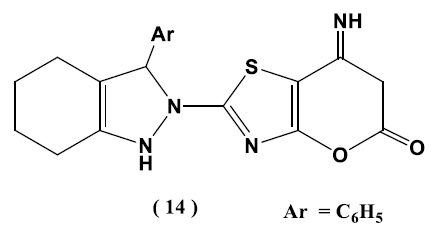


7

Fig. (S6b). 1H-NMR spectrum of compound 7


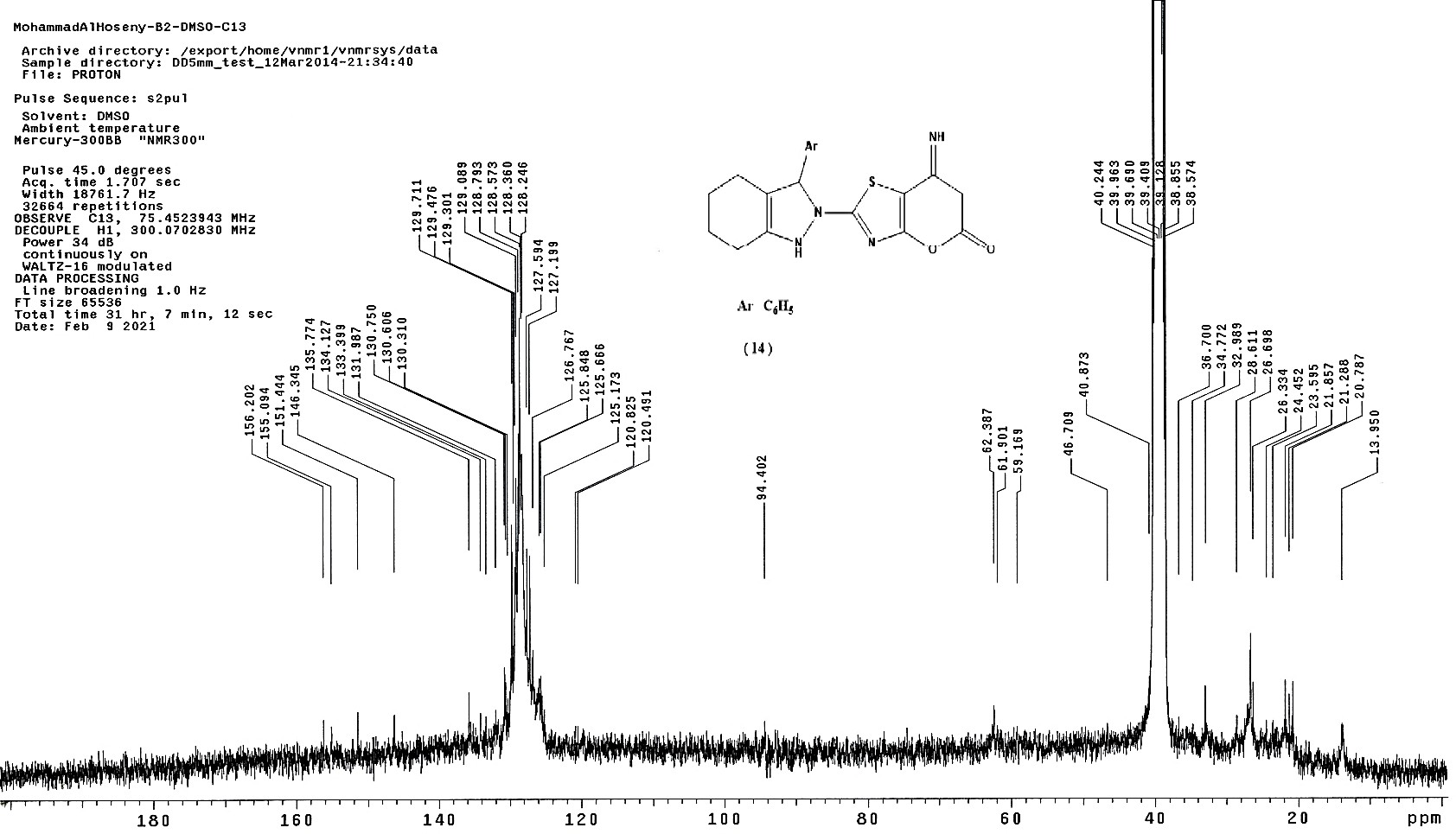

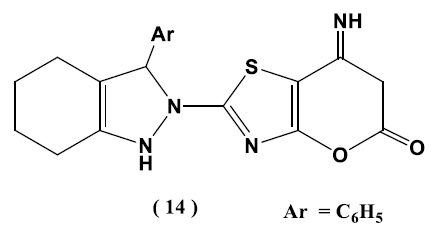


7

Fig. (S6 c).13C-NMR spectrum of compound 7


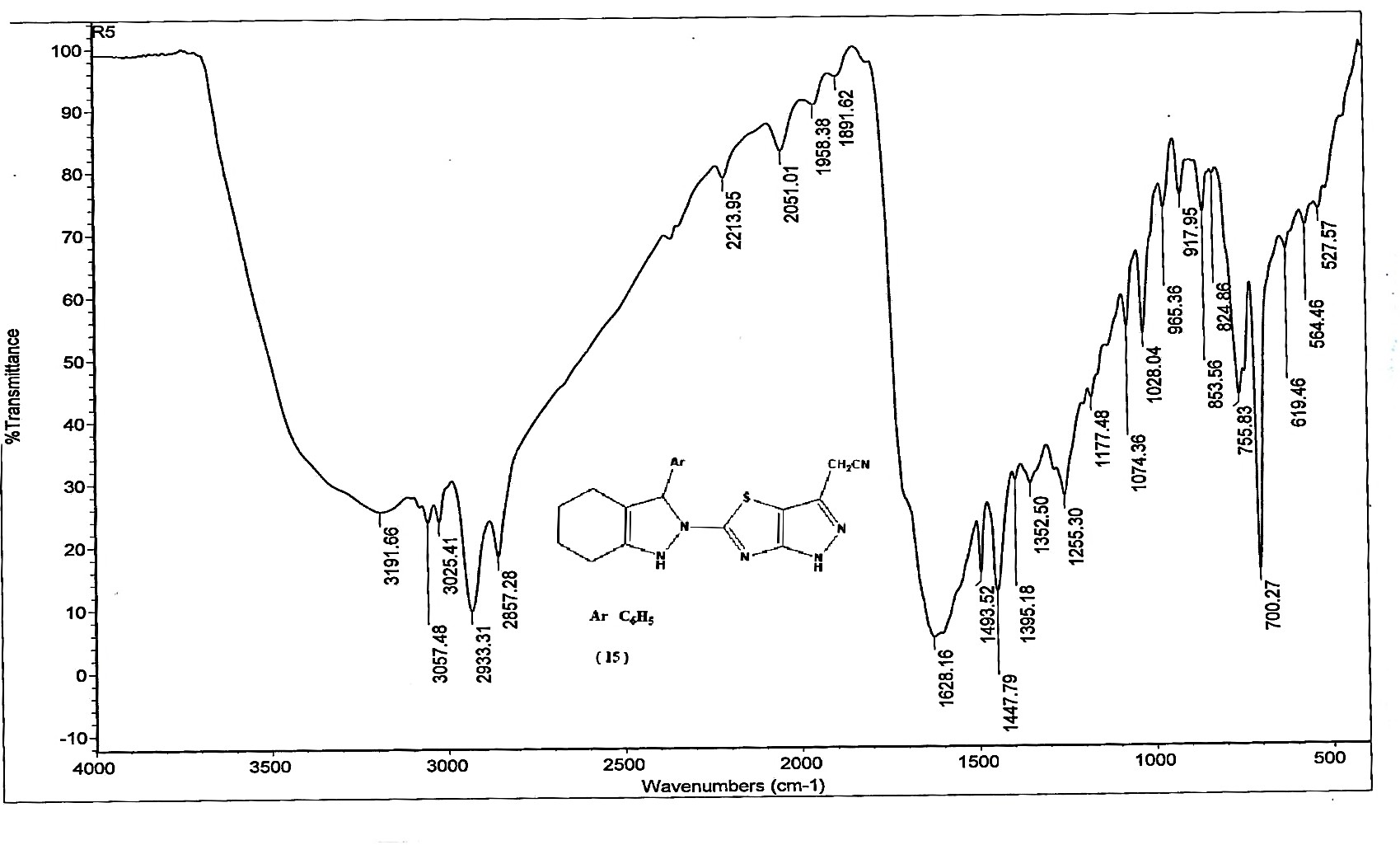

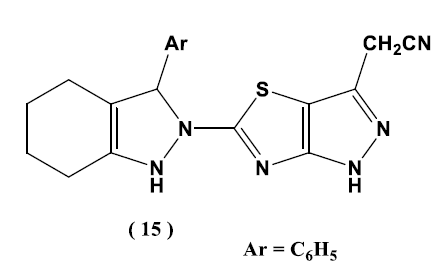


8

Fig. (S7 a). IR spectrum of compound 8


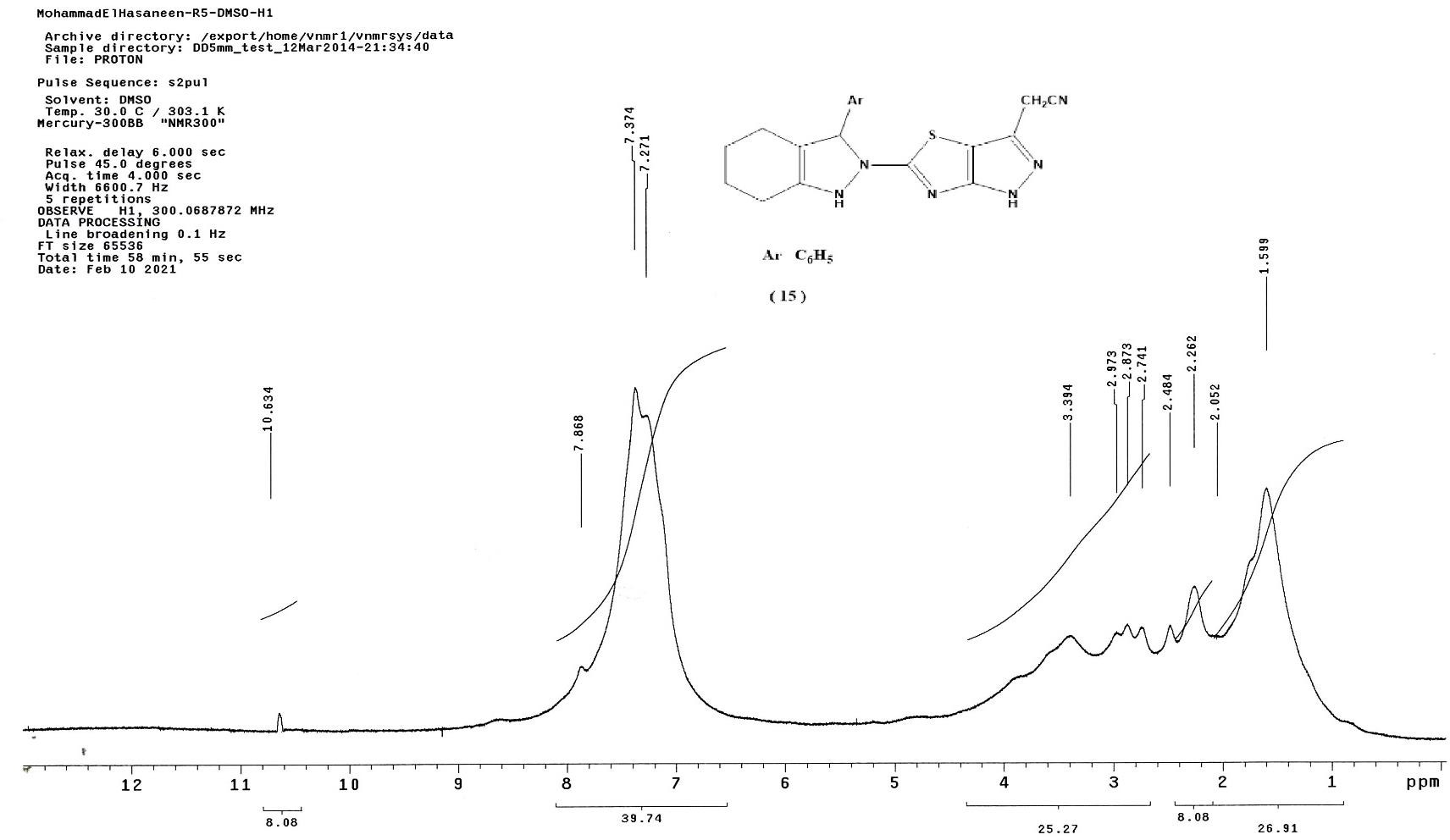

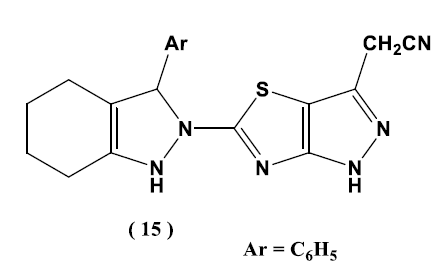


8

Fig. (S7 b). 1H-NMR spectrum of compound 8


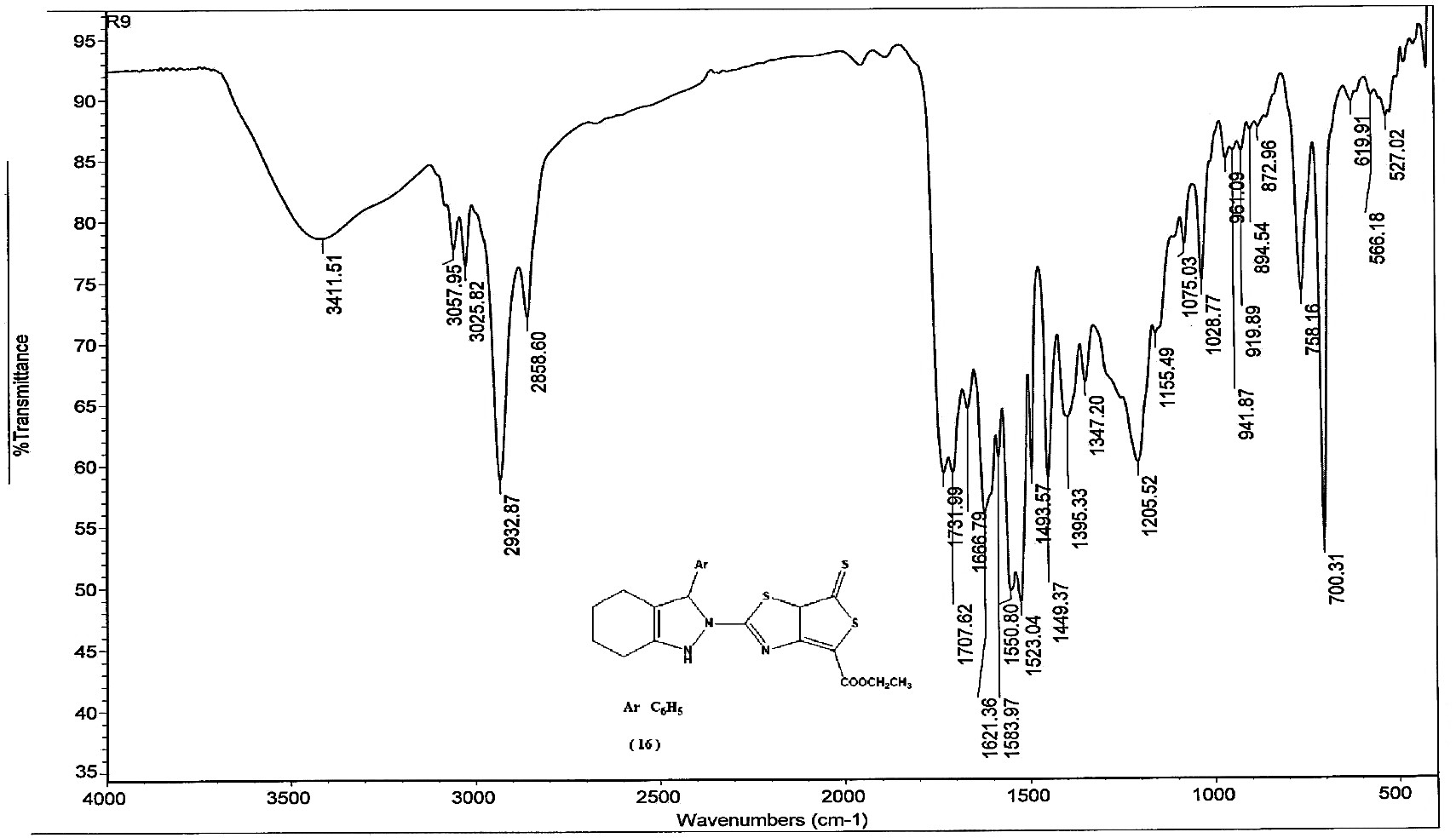

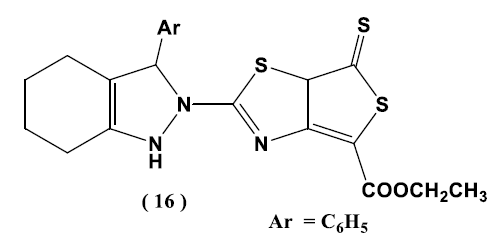


9

Fig. (S8 a).IR spectrum of compound 9


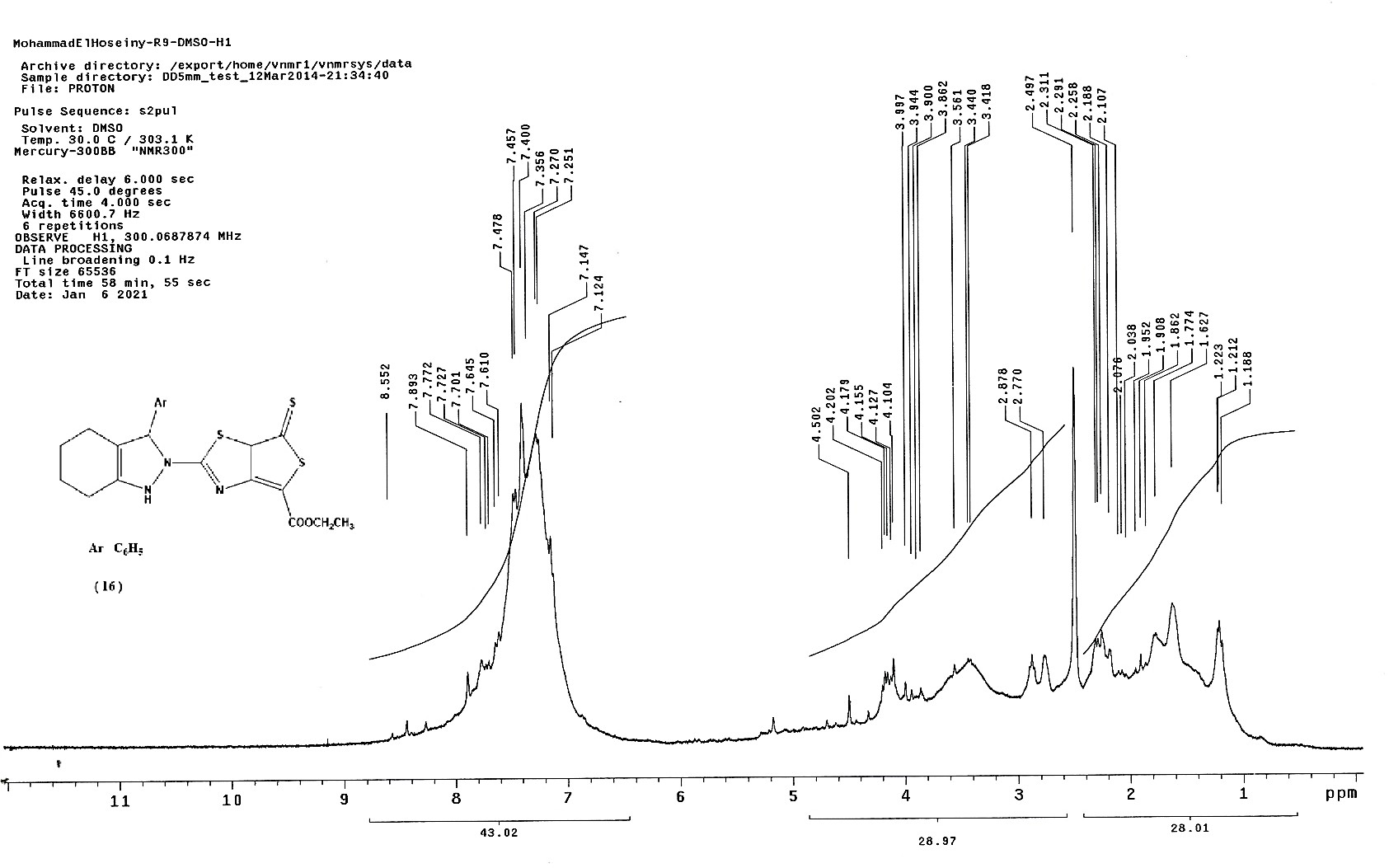

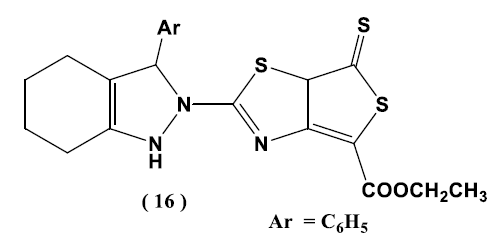


9

Fig. (S8 b). 1H-NMR spectrum of compound 9
